# A comparison of acellular dermal matrices (ADM) efficacy and complication profile in women undergoing implant-based breast reconstruction: a systematic review and network meta-analysis

**DOI:** 10.1186/s12885-024-13359-3

**Published:** 2024-12-31

**Authors:** Sevasti Panagiota Glynou, Sara Sousi, Hannah Cook, Alexander Zargaran, David Zargaran, Afshin Mosahebi

**Affiliations:** 1 London School of Medicine and Dentistry, Queen Mary University of London, London, UK; 2 Faculty of Medicine, Imperial College London, London, UK; 3 Department of Plastic Surgery, University College London, London, UK; 4 Royal Free London NHS Foundation Trust, London, UK; 5 British Association of Aesthetic Plastic Surgeons (BAAPS) Academy, London, UK

**Keywords:** Breast surgery, Reconstructive breast surgery, Acellular dermal matrix, ADM

## Abstract

**Introduction:**

Breast cancer is the leading cause of cancer amongst women in the United Kingdom, with implant-based reconstruction (IBR) using Acellular Dermal Matrices (ADM) gaining popularity for post-mastectomy procedures. This study compares outcomes of different ADMs that are commonly used in women undergoing IBR, this was short and long-term complications.

**Methods:**

A systematic search of MEDLINE, Embase, CENTRAL, and CDSR databases was performed according to the PRISMA guidelines, focusing on women undergoing IBR with FlexHD, AlloDerm, Bovine, or Porcine ADMs. A network meta-analysis (NMA) was also conducted.

**Results:**

A total of 51 studies were captured by the search, of which 27 were included in the network meta-analysis. Alloderm was the most used ADM (54%), followed by Porcine (17%), Bovine (11%), DermAcell (11%), and FlexHD (7%). The mean follow-up was 27.8 months. The complication rates varied. Porcine ADMs had the highest rate of seroma formation (10.3%) and of haematoma formation (2.7%). AlloDerm FD had the highest rate of wound dehiscence (3.1%). Implant failure was highest in AlloDerm FD ADMs (11.8%), followed by Porcine ADMs (11.2%). Infections were most common in Porcine (11.2%) and AlloDerm FD ADMs (11.0%). Capsular contracture was rare across all ADM types, with no significant differences observed. In the NMA, AlloDerm FD showed significantly higher risks of infection, explantation, and wound dehiscence compared to AlloDerm RTU.

**Conclusion:**

The overall complication profiles of ADMs used in IBR are similar, except for the higher risks associated with AlloDerm FD compared to RTU. These findings suggest that the choice of ADM may not significantly impact overall outcomes, except in specific cases like AlloDerm FD. Further high-quality, long-term, double-arm studies are necessary to confirm comparative profile of specific ADM types and to account for potential confounding variables through multivariable regression analysis.

**Supplementary Information:**

The online version contains supplementary material available at 10.1186/s12885-024-13359-3.

## Introduction

Breast cancer is the most prevalent cancer among women globally, and the most frequently diagnosed malignancy, with a worldwide incidence of 2.26 million in 2020^1^. Implant-based reconstruction (IBR) accounts for 37% of immediate reconstructions after mastectomy in the United Kingdom (UK) [2]. The use of Acellular Dermal Matrices (ADM) in breast surgery was first documented in 2002 by Duncan to address implant rippling in revisional cosmetic surgery [3]. Since then, it has acquired increasing popularity in both immediate and delayed breast reconstruction, with an estimated 25–75% of tissue expander reconstructions using ADMs [4]. Based on their source materials, ADMs can be categorised into human, bovine, porcine or synthetic products.

ADM is a biodegradable surgical mesh derived from mammalian tissues (human, porcine, or bovine), subjected to decellularization, resulting in a connective tissue graft that acts as a scaffold. This scaffold is thought to facilitate incorporation into the recipient site and promote revascularization [5].

As implant-based reconstruction has increased in popularity, so has the use of ADM. Various ADM types are well established and widely employed, whilst new ones frequently emerge in the market [6]. They have become popular in direct-to-implant procedures, particularly with pre-pectoral reconstruction, tissue direct-to-implant placement reported to aide in maximising successful outcomes [7].

The reported advantages of using ADM-based breast reconstruction are enhanced soft tissue coverage of the lower pole, increased intraoperative fill volumes, and superior cosmetic results [8]. They are believed to provide structural support to the soft tissue, thereby improving implant positioning and assisting in expanding the lower pole of the breast in dual-plane reconstruction [7], leading to improved aesthetic outcomes and a reduced risk of capsular contracture [9]. However, the complication profile associated with ADM use remains a topic of ongoing debate.

Current reports point to relatively high complication rates, including an elevated risk of seroma, infection, skin necrosis and the need for explanation [10]. In 2021, the U.S. Food and Drug Administration (FDA) issued a safety communication emphasising that ADMs are not approved or cleared specifically for use in IBR. The FDA expressed concerns about the off-label use of ADMs in this context, advising healthcare providers to be well-informed about the potential risks [11].

In particular, the FDA’s analysis of the Mastectomy Reconstruction Outcomes Consortium (MROC) revealed that, two years after surgery, patients who received FlexHD and AlloMax brands of ADM experienced notably higher rates of complications of implant removal, reoperation, and infections, compared to those who received SurgiMend, AlloDerm, or no ADM at all.

Currently, although some studies have compared the outcomes of two or three different ADMs [12–15] conclusive evidence comparing all the most common ADMs in the literature remains limited. Furthermore, in May 2023, Integra issued an immediate market recall of its bovine ADM, SurgiMend 43; due to higher levels of endotoxins were released that exceeded the permitted levels as per the product specifications [16]. 

This systematic review and network meta-analysis aims to address this gap by comparing the most commonly used ADM types in implant-based breast reconstruction internationally [12]. This is defined by short- and long-term complications, rate of infection and implant failure. The ADM types included in this study were AlloDerm (all-type, Freeze-Dried, Ready-To-Use), DermACELL, Bovine (SurgiMend), Flex HD, and Porcine.

## Materials and methods

### Study question

This study aims to compare the operative success of different ADM types that are commonly used in women undergoing implant-based breast reconstruction. This review was registered on PROSPERO [17] with the following reference number: CRD42023400616.

### Literature search

A literature search was conducted supported by the services of the Royal College of Surgeons of England. The databases queried were Ovid MEDLINE, Embase, Cochrane Central Register of Controlled Trials (CENTRAL), and Cochrane Database of Systematic Reviews (CDSR).

The search strategy included a combination of the following terms: Acellular dermal matrix (ADM); Flex HD; AlloDerm; SurgiMend; Braxon; Artia; Strattice; Mammaplasty; Breast implantation; Breast reconstruction; Mastectomy; Breast cancer; Post-operative complications; Treatment outcomes; Quality of life. The search string was limited to studies published in the last 10 years, and the latest search was conducted in February 2023, with the search being re-run in August 2024. Table 1 demonstrates the search string using the Population, Intervention, Comparison, and Outcomes (PICO) methodology.

**Table 1:** Study Population, intervention, comparison, and outcomes (PICO)

**Population(s)**	1) Women undergoing implant-based breast reconstruction with ADM and without autologous flap-based reconstruction
	2) Women undergoing reconstruction using any of the following ADM types: FlexHD, AlloDerm, Strattice, Braxon, DermACELL, Artia, and SurgiMend
	3) Immediate or delayed reconstruction
	4) Unilateral or bilateral reconstruction
**Intervention(s)**	Use of different types of ADM during breast reconstruction procedures (Allografts, and Xenografts)
	The study domain is breast reconstruction following mastectomy for breast cancer treatment or prophylaxis.
	Breast reconstructions specifically studied are implant-based.
**Comparators**	Different types of ADM used during breast reconstruction procedures.
**Outcomes**	Operative success, defined by the following:
	1) complications
	2) implant failure
	3) infections
	4) patient quality of life

### Study selection

Initial studies underwent title and abstract screening, full-text review, and data extraction by two reviewers independently, assessing the suitability and relevance based on the inclusion/exclusion criteria (Table 2) and the described outcomes, respectively. Any disagreement with regards to the study selection was resolved by a third independent reviewer.

**Table 2:** Exclusion criteria

Exclusion Criteria	- secondary reconstructive procedures such as reconstruction revision
	- aesthetic or cosmetic procedures
	- non-implant-based reconstruction, for example, autologous free flaps
	- non-English language
	- animal or cadaveric studies
	- systematic review including papers already present in results
	- revision surgeries

### Study quality

Risk of bias and study quality of the studies was evaluated using the Newcastle-Ottawa Scale (NOS) [18] for observational studies, and the CONSORT 2010 checklist [19] was used for randomised control studies (RCTs). The NOS tool assigns studies a total score out of 9 across the following three categories: selection (out of 4), comparability (out of 2) and outcome (out of 3). Using the CONSORT checklist, each of the 37 items were given a score 0 if the details required had not been / were partially reported and a score of 1 if they had been reported. To determine the overall compliance, the percentage of fulfilled CONSORT checklist items was calculated by summing the scores achieved and dividing it by the total number of checklist items. This was carried out by two reviewers independently, and the scores were correlated.

### Data extraction & network meta-analysis

Study characteristics (author, year of publication, country of origin, study type, number of arms, ADM subcategory), patient demographics and comorbidities, additional therapies, surgical techniques, and surgical outcomes were extracted. The primary outcome of the study was the incidence of the most commonly reported complications associated with each ADM type. These included short term complications (seroma, hematoma, wound dehiscence), long-term complications (capsular contracture, rotation), failure (implant removal), and infection. If there were discrepancies in the extracted data, it was resolved by a third independent reviewer.

Due to the diverse array of ADMs employed in breast reconstruction between different countries and the inadequate reporting of the subtype between studies, ADMs were grouped into 7 ADM subtypes, namely: AlloDerm^®^ FD, AlloDerm^®^ RTU, AlloDerm^®^ Unspecified, DermACell^®^, Flex HD, Porcine - Strattice™, and Bovine – SurgiMend, for the network-meta-analysis. The first five subtypes were human-derived (Allograft), whilst the last two were animal-derived (Xenograft). AlloDerm^®^ Unspecified was created as a new category as some papers did not define the specific type used. Table 3 illustrates the breakdown and corresponding manufacturer of subtypes.

**Table 3:** ADM type categorisation. * unspecified is defined for those studies where AlloDerm is used in a study, however, the type is not specified

**Allografts**	
AlloDerm^®^ FD	LifeCell Corp., Branchburg, New Jersey, USA
AlloDerm^®^ RTU	
AlloDerm^®^ Unspecified*	
DermaCell^®^	Lifenet, Virginia Beach, Virginia, USA
FlexHD^®^	MTF/Ethicon, Somerville, New Jersey, USA
**Xenografts**	
Porcine - Strattice™	LifeCell Corp., Branchburg, New Jersey, USA
Porcine - ARTIA™	Allegran Inc, California, USA
Porcine^®^ - Braxon	QuaMedical B.V., Zuidwolde, The Netherlands
Bovine - SurgiMend^®^	TEI Biosciences, Boston, Massachusetts, USA

The data extracted were exported into standardised Microsoft Excel spreadsheets, by two independent reviewers, any discrepancies were discussed and resolved by a third independent reviewer. Studies that met the inclusion criteria were reported in the qualitative side of the systematic review, and those with two or more arms were included in the quantitative analysis of this study, i.e., the network meta-analysis. No cut off was used for sample size of the study’s arms, as the NMA methodology synthesises direct and indirect evidence, mitigating the impact of smaller studies. The statistical analysis was carried out in R (version 4.0.3) [20] using the “netmeta” package [21].

## Results

**Fig. 1:**
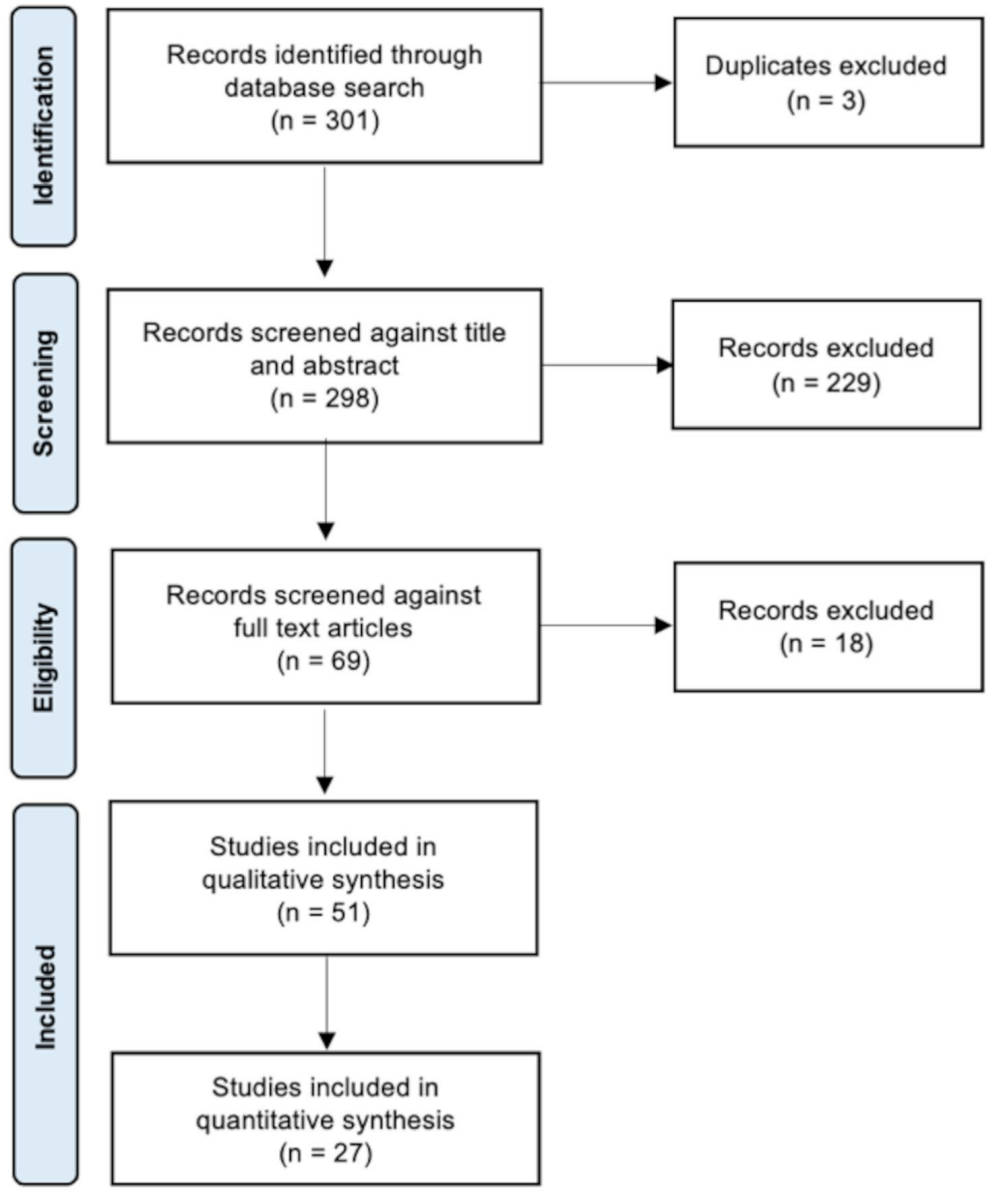
PRISMA flowchart – article screening process [22]

The search string resulted in 51 studies meeting the defined inclusion and exclusion criteria, of which 27 were included for the network meta-analysis. The Kappa score for interrater reliability was 0.93, indicating good inter-reviewer agreement. The review process is illustrated in Fig. 1.

### Study and operative characteristics

In total, there were 7,667 patients and 11,988 breasts, with some studies reporting number of breasts alone, not reporting the number of patients. AlloDerm was the most prevalent ADM used in 54.4% of the arms, followed by Porcine (16%), Bovine and DermACELL (11.1% each), and Flex HD (7.4%). Table 4 provides the breakdown of arms at study level, and the ADM subtype used, alongside patient demographics, and treatment type.

**Table 4:** Patient and study characteristics; where values are not presented it is because they were not recorded. An asterisk (*) under treatment types refers to the value not being specified it is neo-adjuvant or adjuvant treatment

					Comorbidities				Treatment Type			
Study	Country	ADM Type	No. Patients	No. Breasts	Mean Age	Mean BMI	Smoker	Diabetes	Neo-Adjuvant Chemotherapy	Adjuvant Chemotherapy	Neo-Adjuvant Radiotherapy	Adjuvant Radiotherapy
Arnaout et al. (2021) [71]	Canada	DermAcell	33	40	51.4	24.9	3.2%	3.2%	9.7%		20.0%	
		AlloDerm RTU	33	41	47.8	24.9	9.7%	0.0%	12.9%		12.2%	
Asaad et al. (2021) [36]	USA	AlloDerm RTU	36	55	49	26	8.0%	3.0%	28.0%	33.0%		16.0%
		Bovine (SurgiMend)	32	48	50	26	13.0%	16.0%	25.0%	38.0%		19.0%
Baker et al. (2018) [70]	UK	Porcine (Strattice)	40	62	47.8	24.7	12.5%		4.0%		0.0%	
Ball et al. (2017) [72]	UK	Porcine (Strattice)	19	30	44	23	15.8%		36.8%	0.0%	5.3%	3.7%
		Bovine (SurgiMend)	62	89	46.9	23.4	22.6%		29.0%	14.5%	6.5%	20.1%
Bassetto et al. (2022) [68]	Italy	Porcine (Braxon)	18	22	57	24	16.7%	5.6%	66.7%*		68.2%*	
Broyles et al. (2021) [73]	USA	FlexHD	113	187	46.5	25.6	0.0%	1.8%	15.9%	31.9%		14.4%
		AlloDerm RTU	117	197	46.8	24.4	0.9%	5.1%	19.7%	30.8%		16.2%
Buseman et al. (2013) [27]	USA	AlloDerm FD	25	25	48	26.5	28.0%		40.0%	44.0%	0.0%	4.0%
		AlloDerm RTU	9	9	49.6	25.7	33.0%		11.0%	11.0%	0.0%	11.0%
Butterfield et al. (2013) [35]	USA	AlloDerm (Unspecified)	59	89	47.5	26.3	12.0%	5.0%	20.0%	25.0%	6.0%	7.0%
		Bovine (SurgiMend)	222	351	48.6	27	7.0%	1.0%	32.0%	22.0%	6.0%	4.0%
Chang et al. (2017) [7]	USA	FlexHD	18	32	47.6	24.9	16.7%		16.7%	22.2%	33.3%*	
		DermAcell	14	20	54.1	25.7	8.3%		41.7%	25.0%	33.3%*	
		AlloDerm (Unspecified)	15	22	47.5	25.7	0.0%		13.3%	26.7%	20.0%*	
Eichler et al. (2015) [74]	Germany	Bovine (SurgiMend)	57	57	55.4	21.8	14.3%		23.8%*		14.3%*	
Eichler et al. (2017) [75]	Germany	Bovine (SurgiMend)	17	17	49.8	22.6	16.7%	5.6%	55.6%*		50.0%*	
Fakim et al. (2019) [76]	UK	Porcine (Artia)	51	83	42.9	24.7	9.8%	1.9%	5.9%	9.8%	0.0%	1.2%
Frey et al. (2015) [57]	USA	AlloDerm FD		91	49.1	26.5	5.5%	4.4%			3.3%	14.3%
		AlloDerm RTU		164	49.4	24.2	4.3%	4.3%			7.3%	10.4%
Greig et al. (2019) [32]	Canada	DermAcell	36	56	53.1	24.9	0.0%	0.0%		47.2%		19.6%
		AlloDerm (Unspecified)	28	39	52.4	24.3	0.0%	3.0%		50.0%		25.6%
Hanson et al. (2018) [77]	USA	AlloDerm FD	389	532	49.7	26.6	6.2%	5.9%	30.3%		2.3%	
		AlloDerm RTU	316	456	48.6	26.2	2.8%	2.8%	27.8%		3.2%	
Hillberg et al. (2018) [78]	The Netherlands	Porcine (Strattice)	19	28	41.4	22.7	15.8%		26.3%	57.9%	17.9%*	
Hinchcliff et al. (2017) [79]	USA	AlloDerm RTU	15	25	49	25.4	6.7%	13.3%			13.3%	20.0%
Jafferbhoy et al. (2017) [80]	UK	Porcine (Braxon)	64	78	50	25.7	20.3%	3.1%	9.4%		3.1%	
Jeon et al. (2021) [81]	Korea	DermAcell	32	32	44.3	22.9	0.0%	0.0%	31.3%	31.3%		31.3%
Keifer et al. (2016) [26]	USA	AlloDerm (Unspecified)	98	174	48.5	23.4	50.0%	6.0%			71.4%	
Klein et al. (2019) [23]	USA	AlloDerm RTU	17	27	49.9	26.6	7.4%	0.0%	14.8%	33.3%	3.7%	3.7%
		AlloDerm (Unspecified)	3	6	48.7	23.5	33.3%	0.0%	33.3%	0.0%	0.0%	0.0%
Lardi et al. (2014) [82]	UK + Switzerland	Porcine (Strattice)	149	200	48	24.9	16.8%	1.0%	14.1%	43.6%	2.0%	38.3%
Lee et al. (2013) [34]	Korea	AlloDerm (Unspecified)	31	31	43.6	23.5	3.2%	0.0%		22.6%		
Lewis et al. (2015) [25]	USA	AlloDerm FD	60	93	51.9	27	12.0%	1.7%				
		AlloDerm RTU	45	74	55.3	27.3	18.0%	2.2%				
Liu et al. (2014) [15]	USA	AlloDerm (Unspecified)	165	175	48		13.9%	1.8%	17.6%		0.6%	
		FlexHD	97	113	47.4		7.2%	4.1%	14.4%		2.1%	
Lohmander et al. (2019) [83]	UK + Sweden	Porcine (Strattice)	64	66	51.8	23.6				52.0%		20.0%
Loo et al. (2018) [84]	UK	Porcine (Strattice)	670	850	50		33.6%		22.4%	32.1%		19.6%
Mazari et al. (2018) [85]	UK	Porcine (Strattice)	45	54	49.1	24.3	8.9%	0.0%				
		Bovine (SurgiMend)	37	43	49.6	24.1	8.1%	2.7%				
Mendenhall et al. (2015) [86]	USA	AlloDerm (Unspecified)	59	94								
Mendenhall et al. (2017) [87]	USA	AlloDerm FD	57	91	48	27	0.0%	5.3%	14.0%	22.8%		17.6%
Michelotti et al. (2013) [13]	USA	FlexHD		61	50.7	26.3	2.0%	0.0%	41.0% *		25.0%*	
		AlloDerm (Unspecified)		49	49.9	26.8	10.0%	4.0%	57.0%*		24.0%*	
		DermAcell		110	50.8	26.3	15.0%	6.0%	54.0%*		12.0%*	
Ohkuma et al. (2013) [88]	USA	Bovine (SurgiMend)	64	94	50.9	26	39.0%	8.5%	31.0%	36.0%	8.5%	17.0%
Parikh et al. (2018) [67]	USA	AlloDerm RTU	17	28	51.9	26.4	5.8%		23.5%*			
Parikh et al. (2018) [89]	USA	AlloDerm FD	612	910	49.5	28.5	20.3%	7.8%	29.9%	42.8%		35.5%
		AlloDerm RTU	673	1,129	49.6	28.1	15.2%	6.5%	22.6%	29.9%		33.1%
Park et al. (2021) [90]	Korea	AlloDerm FD	26	26	43.4	23.1	0.0%	7.7%	0.0%	61.5%	0.0%	1.0%
		AlloDerm RTU	52	52	47.7	23.8	1.9%	5.8%	5.7%	57.6%	1.9%	3.8%
Pittman et al. (2017) [24]	USA	DermAcell	30	50	47.7	25.8	3.0%	0.0%	27.0%	17.0%	2.0%	10.0%
		AlloDerm RTU	28	50	46	24.1	0.0%	0.0%	11.0%	25.0%	2.0%	10.0%
Powers et al. (2021) [30]	USA	DermAcell	38	69	45	25.6		2.6%	21.1%*		31.6%*	
		AlloDerm (Unspecified)	41	65	49	25.4		4.9%	24.4%*		29.3%*	
Ranganathan et al. (2015) [12]	USA	FlexHD	186	315	47.2	26.3	4.3%	2.2%	8.6%*		3.8%*	
		AlloDerm (Unspecified)	123	206	47.4	26.5	8.9%	6.5%	15.4%*		6.5%*	
Ricci et al. (2016) [91]	USA	AlloDerm (Unspecified)	400	578	48.3	26.1	6.1%	3.5%	13.3%	32.0%	9.1%	18.7%
		Bovine (SurgiMend)	240	374	47.4	24.8	1.9%	1.3%	9.1%	29.4%	3.7%	17.4%
Salzberg et al. (2013) [92]	USA	Porcine (Strattice)	54	105			9.3%		7.4%*		5.7%*	
Sigalove et al. (2022) [28]	USA	AlloDerm (Unspecified)	128	249	51.2	29.9		21.9%	28.1%	1.6%	1.6%	6.4%
Sinnott et al. (2021) [93]	USA	Porcine (Strattice)	369	592	52.7	28.7	7.6%	5.1%	13.3%	19.0%		
Sobti et al. (2016) [94]	USA	FlexHD	101	170	49.4	25.6	25.7%		37.6%*		5.9%	21.8%
		AlloDerm FD	41	70	49.7	25.9	31.8%		43.2%*		8.3%	24.2%
		AlloDerm RTU	91	154	49.7	25.9	31.8%		43.2%*		8.3%	24.2%
Swisher et al. (2022) [95]	USA	DermAcell	13	25	48.1	28	15.4%	0.0%	40.0%*		24.0%*	
		AlloDerm (Unspecified)	61	103	49.3	27.8	16.4%	4.9%	53.4%*		19.4%*	
Tierney et al. (2021) [29]	USA	AlloDerm RTU	31	55	51.1	28.5	0.0%	12.9%	90.9%	0.0%	9.1%	0.0%
Wang et al. (2021) [66]	China	Bovine - SurgiMend	44	44	37.5	21.7	6.8%	0.0%	52.3%*		6.8%*	
Weichman et al. (2013) [96]	USA	AlloDerm FD	58	90	49.1	26.6	6.7%		13.3%	26.7%	3.3%	14.4%
		AlloDerm RTU	64	105	49.9	24.9	5.7%		12.4%	34.3%	8.6%	5.8%
Widmyer et al. (2019) [31]	USA	AlloDerm FD	94	151	49.7	26.5	12.6%	2.7%				
		AlloDerm RTU	142	227	49.7	26.5	12.8%	5.7%				
Wilson et al. (2022) [69]	UK	Porcine (Strattice)	117	169	54	24.5	6.8%	2.6%			5.1%	6.8%
Yuen et al. (2014) [33]	USA	AlloDerm FD	51	96	50.5	30.3		8.0%	22.0%	8.0%	10.0%	10.0%
		AlloDerm RTU	52	100	51.2	30.2		6.0%	19.0%	13.0%	4.0%	10.0%
Zenn et al. (2016) [37]	USA	DermAcell	70	119								
		AlloDerm RTU	70	130								

### Study quality

Out of the 51 studies included for analysis, 43 were assessed using the Newcastle-Ottawa scale (NOS) and 8 using the CONSORT Checklist. Using the NOS tool, the quality of the studies was appraised by assessing the selection, comparability, and outcome. The average score was 8 out of 9 across 43 studies. The reported follow-up time was variable, with a mean follow-up time of 27.8 months. Klein et al. [23], Pittman et al. [24], Lewis et al. [25], Keifer et al. [26], Buseman et al. [27], and Michelotti et al. [13] did not report follow-up time. Eight studies that had two arms reported different follow-up time for each arm, namely these were: Sigalove et al. [28], Tierney et al. [29], Powers et al. [30], Widmyer et al. [31], Greig et al. [32], Yuen et al. [33], Lee et al. [34], Butterfield et al. [35]. Powers et al. reported a three-fold difference in follow-up time between the two arms; the Alloderm patients were followed up for 29.4 months and the DermACELL treated ones for 10.1 months [30].

For assessing the quality of RCTs, the CONSORT 2010 checklist was used. The average score was 33 out of 37 across the eight studies. The majority of points were lost in the results section. Supplementary Tables 1 and Supplementary Table 2 illustrate the quality appraisal at study level for the NOS and CONSORT tool, respectively.

### Patient characteristics

Age and BMI was recorded by most authors; across the 51 studies the average age was 48.9 years and the mean BMI value 25.6. Comorbidities included smoking status and diabetes, they were reported in 88% and 71% of the included studies, respectively. Across the selected studies the smoking and diabetes rate was 11.7% and 4.2%, respectively. Half of the studies reported whether the mastectomy was nipple or skin sparing, with an average of 33.9% and 56.6% respectively. Majority (88%) of studies reported whether the operation was immediate or delayed, with 91% of them being immediate. Significant variability in the proportion of patients receiving neoadjuvant and adjuvant chemotherapy or radiotherapy across studies was observed. For instance, neoadjuvant chemotherapy ranged from 0 to 66.7%, reflecting differences in clinical practices and patient selection criteria. Similarly, the use of adjuvant radiotherapy showed substantial variability, with some studies reporting rates as high as 57.9%, potentially influencing the comparability of outcomes across studies. Additionally, some studies did not distinguish on whether the treatment type was adjuvant or neo-adjuvant [12, 13, 30, 66–68, 74, 75, 78, 92, 94, 95].

Table 4 illustrates the patient characteristics and Table 5 the surgical technique at a study and arm level the patient characteristics.

**Table 5:** Surgery techniques of each study; NR: not reported

Study	Avg / Median Follow-up Period	ADM Type	Nipple Sparring	Skin Sparring	Immediate	Delayed	Plane
Arnaout et al. (2021) [71]	6 months	DermACELL	47.5%	52.5%	100%	0%	Subpectoral
		AlloDerm RTU	55.3%	44.7%	100%	0%	Subpectoral
Asaad et al. (2021) [36]	36 months	AlloDerm RTU	11.0%	89.0%	100%	0%	Dual
		Bovine (SurgiMend)	4.0%	92.0%	100%	0%	Dual
Baker et al. (2018) [70]	9.2 months	Porcine (Strattice)	NR		100%	0%	Prepectoral (69%)
							Subpectoral (31%)
Ball et al. (2017) [72]	14 months	Porcine (Strattice)	NR		100%	0%	Dual
		Bovine (SurgiMend)	NR		100%	0%	Dual
Bassetto et al. (2022) [68]	28 months	Porcine (Braxon)	NR		NR		NR
Broyles et al. (2021) [73]	12 months	FlexHD	39.8%	60.2%	100%	0%	Prepectoral (20.3%)
							Subpectoral (79.7%)
		AlloDerm RTU	47.0%	53.0%	100%	0%	Prepectoral (20.8%)
							Subpectoral (79.2%)
Buseman et al. (2013) [27]	NR	AlloDerm FD	NR		NR		NR
		AlloDerm RTU	NR		NR		NR
Butterfield et al. (2013) [35]	AlloDerm: 39 months SurgiMend: 16 months	AlloDerm (Unspecified)	NR		100%	0%	Subpectoral
		Bovine (SurgiMend)	NR		100%	0%	Subpectoral
Chang et al. (2017) [7]	15 months	FlexHD	26.7%		100%	0%	Subpectoral
		DermACELL	0.0%		100%	0%	Subpectoral
		AlloDerm (Unspecified)	22.2%		100%	0%	Subpectoral
Eichler et al. (2015) [74]	NR	Bovine (SurgiMend)	NR		NR		NR
Eichler et al. (2017) [75]	NR	Bovine (SurgiMend)	NR		100%	0%	NR
Fakim et al. (2019) [76]	9 months	Porcine (Artia)	57.8%	27.7%	100%	0%	Dual
							Prepectoral
Frey et al. (2015) [57]	NR	AlloDerm FD	27.5%		100%	0%	Subpectoral
		AlloDerm RTU	51.8%		100%	0%	Subpectoral
Greig et al. (2019) [32]	18 months	DermACELL	34.5%	65.5%	100%	0%	Subpectoral
		AlloDerm (Unspecified)	23.1%	76.9%	100%	0%	Subpectoral
Hanson et al. (2018) [77]	39.7 months	AlloDerm FD	8.1%	96.4%	100%	0%	NR
		AlloDerm RTU	14.5%	98.2%	100%	0%	
Hillberg et al. (2018) [78]	12 months	Porcine (Strattice)	7.1%	17.9%	100%	0%	Dual
Hinchcliff et al. (2017) [79]	12 months	AlloDerm RTU	NR		100%	0%	Subpectoral
Jafferbhoy et al. (2017) [80]	10 months	Porcine (Braxon)	NR		100%	0%	Prepectoral
Jeon et al. (2021) [81]	30 months	DermACELL	53.1%	43.8%	50%	50%	Subpectoral
Keifer et al. (2016) [26]	2 months	AlloDerm RTU	40.7%	62.3%	NR		NR
Klein et al. (2019) [23]	NR	AlloDerm RTU	7.4%		100%	0%	Subpectoral
		AlloDerm (Unspecified)	0.0%		100%	0%	Subpectoral
Lardi et al. (2014) [82]	22 months	Porcine (Strattice)	NR		100%	0%	Subpectoral
Lee et al. (2013) [34]	16 months	AlloDerm (Unspecified)	48.4%	51.6%	100%	0%	Dual
Lewis et al. (2015) [25]	NR	AlloDerm FD	NR		NR		NR
		AlloDerm RTU	NR				
Liu et al. (2014) [15]	6.4 months	AlloDerm (Unspecified)		90.3%	100%	0%	Dual
		FlexHD		87.6%	100%	0%	Dual
Lohmander et al. (2019) [83]	6 months	Porcine (Strattice)	40.0%		100%	0%	Dual
Loo et al. (2018) [84]	29 months	Porcine (Strattice)	3.7%	9.4%	100%	0%	Dual
Mazari et al. (2018) [85]	12–60 months	Porcine (Strattice)	53.7%	31.5%	100%	0%	Dual
		Bovine (SurgiMend)	41.9%	41.9%	100%	0%	Dual
Mendenhall et al. (2015) [86]	NR	AlloDerm (Unspecified)	NR		100%	0%	NR
Mendenhall et al. (2017) [87]	3–24 months	AlloDerm FD	NR		100%	0%	NR
Michelotti et al. (2013) [13]	NR	FlexHD	NR		90%	10%	NR
		AlloDerm (Unspecified)	NR		90%	10%	NR
		DermACELL	NR		95%	5%	NR
Ohkuma et al. (2013) [88]	17 months	Bovine (SurgiMend)	NR		NR		Dual
Parikh et al. (2018) [67]	at least 3 months	AlloDerm RTU	60.7%	39.3%	100%	0%	Prepectoral
							Submuscular
Parikh et al. (2018) [89]	at least 24 months	AlloDerm FD	1.0%		100%	0%	Dual
		AlloDerm RTU	21.4%		100%	0%	Dual
Park et al. (2021) [90]	at least 12 months	AlloDerm FD	19.2%	69.2%	100%	0%	Dual
		AlloDerm RTU	28.8%	63.5%	100%	0%	Dual
Pittman et al. (2017) [24]	NR	DermACELL	NR		100%	0%	Dual
		AlloDerm RTU	NR		100%	0%	Dual
Powers et al. (2021) [30]	DermACELL: 10 months AlloDerm 29 months	DermACELL	86.8%		100%	0%	Prepectoral
		AlloDerm (Unspecified)	85.4%		100%	0%	Prepectoral
Ranganathan et al. (2015) [12]	20 months	FlexHD	NR		93.20%	4.20%	NR
		AlloDerm (Unspecified)	NR				NR
Ricci et al. (2016) [91]	19 months	AlloDerm (Unspecified)	NR		100%	0%	Dual
		Bovine (SurgiMend)	NR		100%	0%	Dual
Salzberg et al. (2013) [92]	41 months	Porcine (Strattice)	NR		100%	0%	Dual
Sigalove et al. (2022) [28]	42 months	AlloDerm (Unspecified)	33.7%	35.7%	100%	0%	Prepectoral
Sinnott et al. (2021) [93]	18 months	Porcine (Strattice)			100%	0%	Prepectoral
Sobti et al. (2016) [94]	NR	FlexHD	NR		100%	0%	NR
		AlloDerm FD	NR		100%	0%	NR
		AlloDerm RTU	NR		100%	0%	NR
Swisher et al. (2022) [95]	DermACELL: 4.6 months AlloDerm: 5.8months	DermACELL	NR		100%	0%	NR
		AlloDerm (Unspecified)	NR		100%	0%	NR
Tierney et al. (2021) [29]	22.3 months	AlloDerm RTU	32.7%	67.3%	100%	0%	Prepectoral (90.9%)
							Subpectoral (9.1%)
Wang et al. (2021) [66]	11 months	Bovine (SurgiMend)	95.5%	4.5%	100%	0%	Subpectoral
Weichman et al. (2013) [96]	NR	AlloDerm FD	27.7%		100%	0%	Dual
		AlloDerm RTU	49.2%		100%	0%	Dual
Widmyer et al. (2019) [31]	at least 12 months	AlloDerm FD	NR		80%	20%	Subpectoral
		AlloDerm RTU	NR		81.50%	18.50%	Subpectoral
Wilson et al. (2022) [69]	62 months	Porcine (Strattice)	15.0%		100%	0%	Subpectoral
Yuen et al. (2014) [33]	AlloDerm FD: 15.2 months AlloDerm RTU: 9.6 months	AlloDerm FD			100%	0%	Dual
		AlloDerm RTU	NR		100%	0%	Dual
Zenn et al. (2016) [37]	6 to 24 months	DermACELL	NR		100%	0%	NR
		AlloDerm RTU	NR		100%	0%	NR

### Complication rates

For short term complications, the occurrence of seroma was reported in 849 (7.1%) breasts, hematoma in 197 (1.6%) breasts, and wound dehiscence in 195 (1.6%) breasts. For long term complications, capsular contraction was reported in 92 (0.8%) and rotation in 6 (0.1%) breasts. There was no data in rippling and skin necrosis in the selected studies, despite forming part of this review’s primary outcomes. Failure was characterised by removal and/or explantation in 792 (6.6%) breasts and infection (major and minor) in 1,062 (8.9%) of breasts. Table 6 provides the breakdown across all 51 studies at ADM subtype level.

**Table 6:** Incidence of the complications, and variables recorded across all studies at ADM Type level, where incidence is defined per breast supplementary tables

			Short Term Complications			Long Term Complications		Failure	Infection
ADM Type	Patients	Breasts	Seroma (%)	Haematoma (%)	Wound Dehiscence (%)	Capsular Contracture (%)	Rotation (%)	Removal / Explantation (%)	Infection (%)
**AlloDerm**	**4,432**	**7,133**	**469 (6.6%)**	**95 (1.3%)**	**125 (1.8%)**	**25 (0.4%)**	**4 (0.1%)**	**574 (8.0%)**	**645 (9.0%)**
AlloDerm FD	1,413	2,175	166 (7.6%)	18 (0.8%)	68 (3.1%)	1 (0.05%)	0 (0.0%)	256 (11.8%)	239 (11.0%)
AlloDerm RTU	1,808	3,078	217 (7.1%)	41 (1.3%)	43 (1.4%)	3 (0.1%)	1 (0.04%)	231 (7.5%)	202 (6.6%)
AlloDerm Unspecified	1,211	1,880	85 (4.5%)	36 (1.9%)	15 (0.8%)	21 (1.1%)	3 (0.2%)	87 (4.6%)	204 (10.8%)
**Porcine**	**1**,**679**	**2**,**339**	**242 (10.3%)**	**63 (2.7%)**	**58 (2.5%)**	**59 (2.5%)**	**0 (0.0%)**	**120 (5.1%)**	**259 (11.1%)**
Porcine - Artia	51	83	6 (7.2%)	1 (1.2%)	0 (0.0%)	0 (0.0%)	0 (0.0%)	2 (2.4%)	0 (0.0%)
Porcine - Braxon	82	100	18 (18.0%)	6 (6.0%)	1 (1.0%)	3 (3.0%)	0 (0.0%)	13 (13.0%)	30 (30.0%)
Porcine - Strattice	1,546	2,156	217 (10.1%)	56 (2.6%)	57 (2.6%)	56 (2.6%)	0 (0.0%)	105 (4.9%)	229 (10.6%)
**Bovine - SurgiMend**	**775**	**1**,**117**	**71 (6.4%)**	**15 (1.3%)**	**4 (0.3%)**	**0 (0.0%)**	**0 (0.0%)**	**52 (4.7%)**	**75 (6.7%)**
**Flex HD**	**515**	**878**	**45 (5.1%)**	**19 (2.2%)**	**4 (0.5%)**	**0 (0.0%)**	**0 (0.0%)**	**22 (2.5%)**	**58 (6.6%)**
**DermACELL**	**266**	**521**	**23 (4.4%)**	**6 (1.1%)**	**4 (0.8%)**	**8 (1.5%)**	**2 (0.4%)**	**24 (4.5%)**	**25 (4.8%)**
**Total**	**7**,**667**	**11**,**988**	**849 (7.1%)**	**197 (1.6%)**	**195 (1.6%)**	**92 (0.8%)**	**6 (0.1%)**	**792 (6.6%)**	**1**,**062 (8.9%)**

Seroma was most prevalent in across all Porcine ADMs at 10.3%, with Braxon type being the highest at 18.3%, the lowest rates were observed by DermACELL at 4.4%. Across all types of ADM, Porcine had the highest hematoma rates at 2.7%, and when looking at subtype Braxon was at 6%, however, this is also due to the low number of breasts in that subtype (*n* = 100). At an overall ADM type Porcine had the highest wound dehiscence rate at 2.5%, while at a subtype level this was exhibited by AlloDerm FD at 3.1%.

Long term complications rates were low with Porcine having the highest rate of capsular contracture at 2.5% overall; while Bovine and Flex HD had no cases of capsular contraction or rotation. Infection rates were highest in reconstructions using porcine ADMs at 11.1%, followed by AlloDerm at 9%. While at ADM subtype the rates were higher, at 30% for Braxon and 11% for AlloDerm FD.

### Network meta-analysis

27 studies were included in the meta-analysis, where the complication, infection, and failure rates were reported. The rates were compared overall at ADM subtype level, and then split at surgical plane, i.e., dual versus pectoral. The forest plots for each reported outcome, and the comparison of each ADM may be seen in Supplementary Fig. 1.

### Seroma

In comparing the relative risks (RRs) of seroma formation across all ADM types with AlloDerm RTU as the reference type, Flex HD, Bovine – SurgiMend and Porcine – Strattice have increased RR, while AlloDerm FD and DermACELL have decreased RR. However, none reached statistical significance (Supplementary Fig. 1A).

**Fig. 2:**
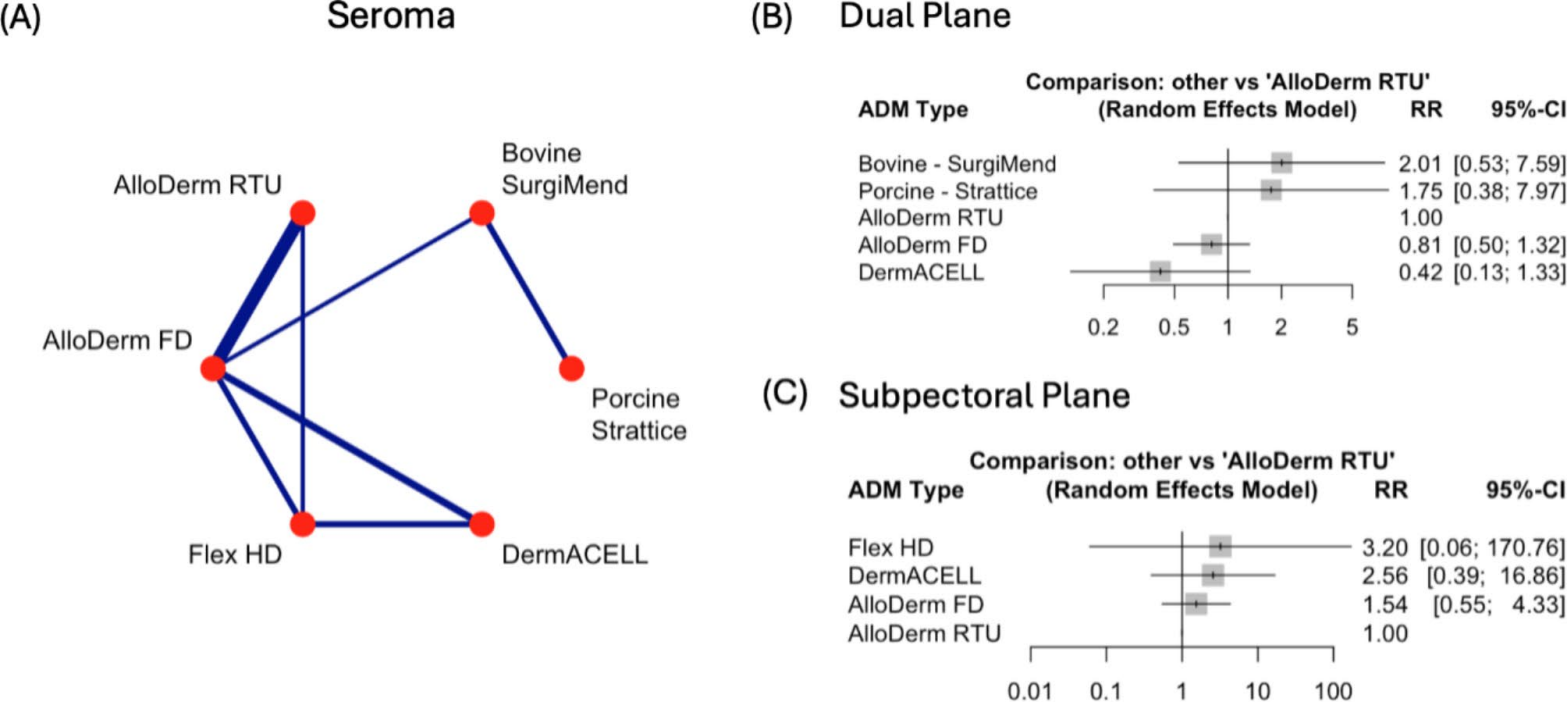
Comparison of Seroma rates across Acellular Dermal Matrices (ADMs) and surgical planes. **(A)** Network plot of pairwise comparisons between ADMs for seroma formation. Line thickness reflects the number of studies. **(B)** Forest plot of risk ratios (RR) and 95% confidence intervals (CI) comparing ADMs in the dual plane, with AlloDerm RTU as the reference. **(C)** Forest plot of RR and 95% CI for ADMs in the subpectoral plane, with AlloDerm RTU as the reference

When looking at the surgical plane, Bovine – SurgiMend had a two-fold increase in seroma risk (RR = 2.01, 95% CI: 0.53–7.59), however, it was not statistically significant (*p* = 0.306). While DermACELL had a decrease in risk of 58% in comparing to AlloDerm RTU (RR = 0.42, 95%CI: 0.13, 1.33), however, this also failed to reach statistical significance (Fig. 2B).

At the subpectoral plane, no comparison reached statistical significance either. It should be noted there were also no comparisons for Porcine – Strattice and Bovine – SurgiMend (Fig. 2C).

### Haematoma

The incidence rate of haematomas was low for all ADM types, when comparing all ADM subtypes with each other no significance was observed with any comparison (Supplementary Fig. 1B). Bovine – SurgiMend versus Porcine – Strattice treated patients had a decreased risk in hematoma by 79% (RR = 0.21, 95% CI: 0.04–1.02, *p* = 0.052).

**Fig. 3:**
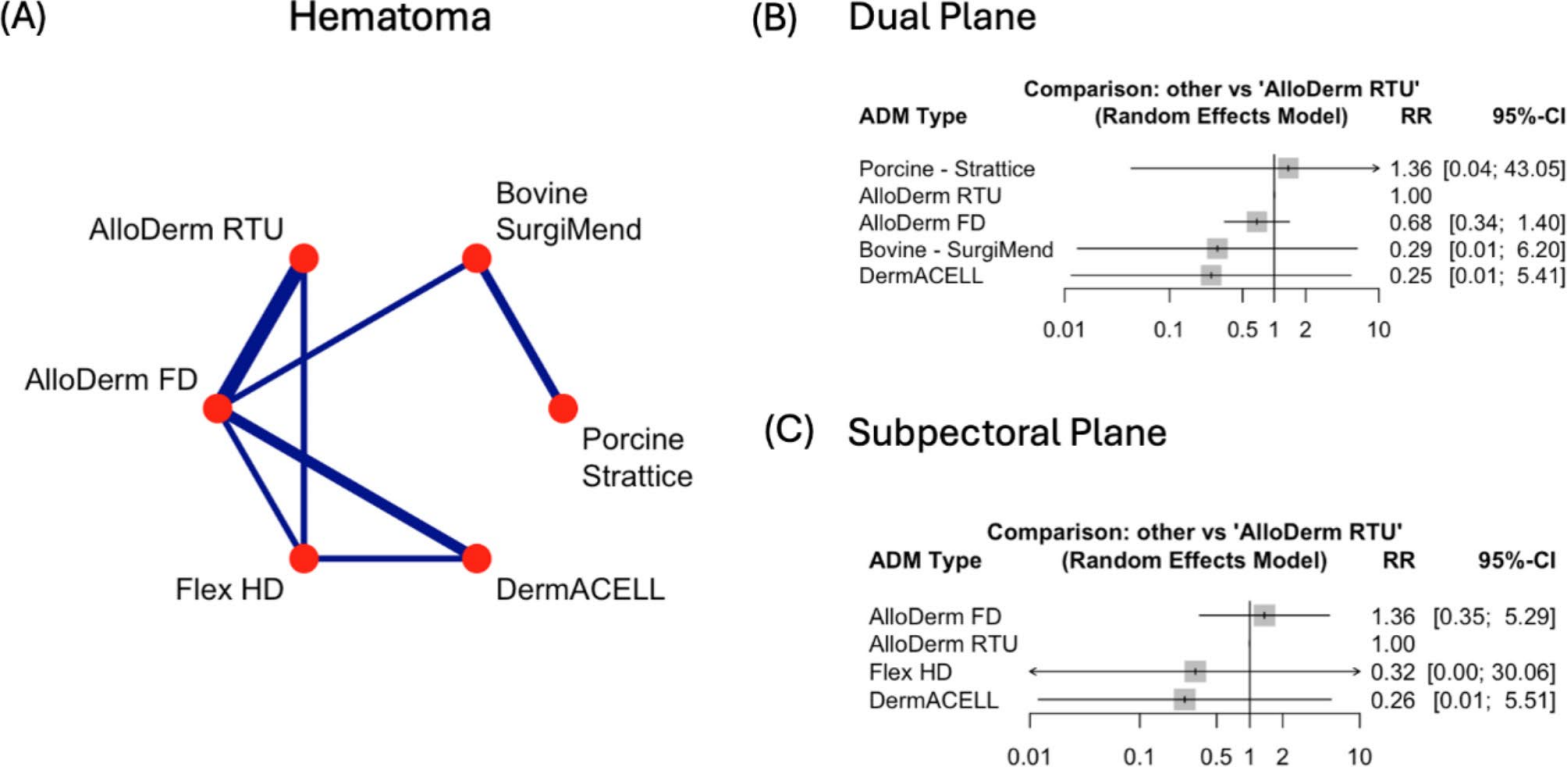
Comparison of Hematoma rates across Acellular Dermal Matrices (ADMs) and surgical planes. **(A)** Network plot of pairwise comparisons between ADMs for seroma formation. Line thickness reflects the number of studies. **(B)** Forest plot of risk ratios (RR) and 95% confidence intervals (CI) comparing ADMs in the *dual plane*, with AlloDerm RTU as the reference. **(C)** Forest plot of RR and 95% CI for ADMs in the *subpectoral plane*, with AlloDerm RTU as the reference

When comparing at surgical plane level, there was no significant differences seen in RR across each ADM type (Fig. 3B, C).

### Wound dehiscence

**Fig. 4:**
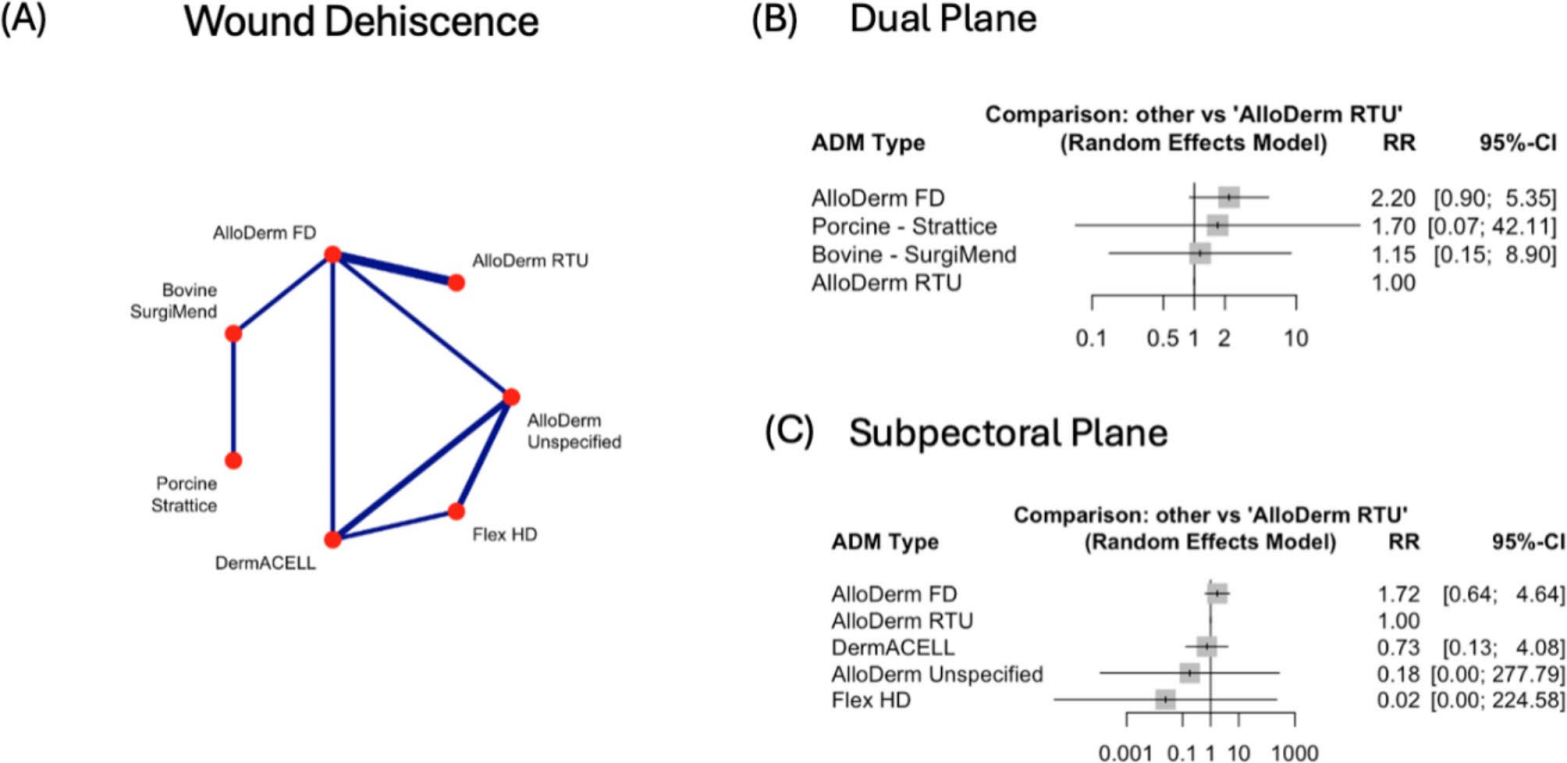
Comparison of Wound Dehiscence rates across Acellular Dermal Matrices (ADMs) and surgical planes. **(A)** Network plot of pairwise comparisons between ADMs for seroma formation. Line thickness reflects the number of studies. **(B)** Forest plot of risk ratios (RR) and 95% confidence intervals (CI) comparing ADMs in the *dual plane*, with AlloDerm RTU as the reference. **(C)** Forest plot of RR and 95% CI for ADMs in the *subpectoral plane*, with AlloDerm RTU as the reference

The comparison of wound dehiscence rates between AlloDerm RTU versus AlloDerm FD, revealed a decrease in risk of wound dehiscence occurring by 48% (RR = 0.52, 95%CI: 0.35–0.79, *p* = 0.002). There was no other statistically significant difference in overall risk (Supplementary Fig. 1C). Similarly, at dual and subpectoral plane level, no significant differences were observed (Fig. 4B, C). However, it should be noted that AlloDerm FD in comparison to AlloDerm RTU had increased risk of wound dehiscence, in both plane types (dual: RR = 2.20, subpectoral: RR = 1.72).

### Capsular contracture

**Fig. 5:**
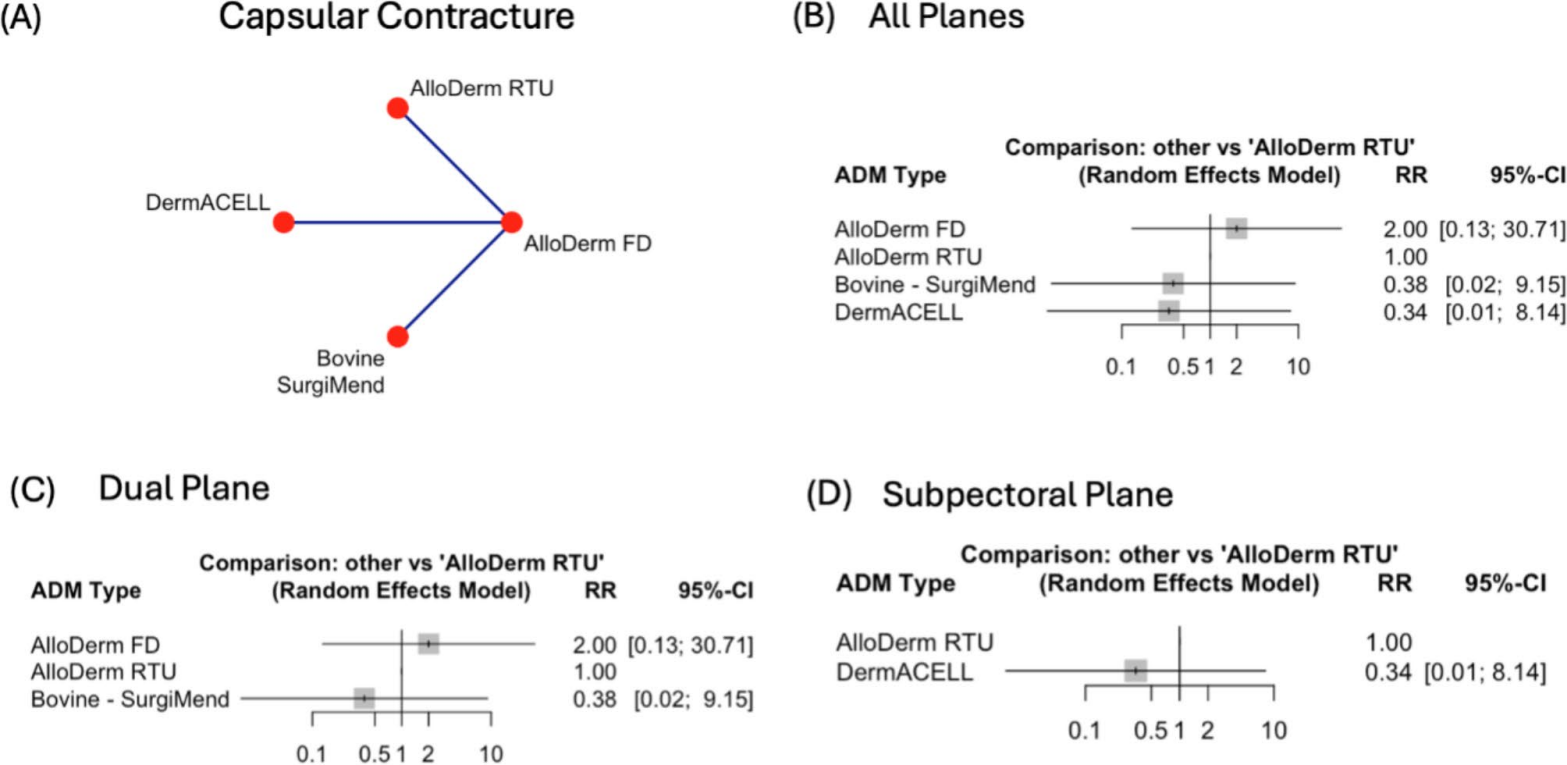
Comparison of Capsular Contracture rates across Acellular Dermal Matrices (ADMs) and surgical planes. **(A)** Network plot of pairwise comparisons between ADMs for seroma formation. Line thickness reflects the number of studies. **(B)** Forest plot of risk ratios (RR) and 95% confidence intervals (CI) comparing ADMs across both planes, with AlloDerm RTU as the reference. **(C)** Forest plot of RR and 95% CI for ADMs in the *dual plane*, with AlloDerm RTU as the reference. **(D)** Forest plot of RR and 95% CI for ADMs in the *subpectoral plane*, with AlloDerm RTU as the reference

Capsular contracture was infrequently reported in the studies, and where it was reported, rates were typically low, leading to wider confidence intervals. Only three of the studies that were included in the network meta-analysis reported capsular contracture as outcome, with two being dual plane and one subpectoral. Additionally, there were no comparisons with Flex HD and Porcine – Strattice. There was no statistically significant difference when comparing the subtypes, at overall and plane level (Fig. 5B-D).

### Removal / explantation

The rates of breast explantation when comparing AlloDerm FD to AlloDerm RTU, exhibited an increase in risk. AlloDerm FD had a 38% increase (RR = 1.38, 95% CI: 1.16–1.63, *p* < 0.001). This was the only statistically significance observed across all planes (Supplementary Fig. 1E).

**Fig. 6:**
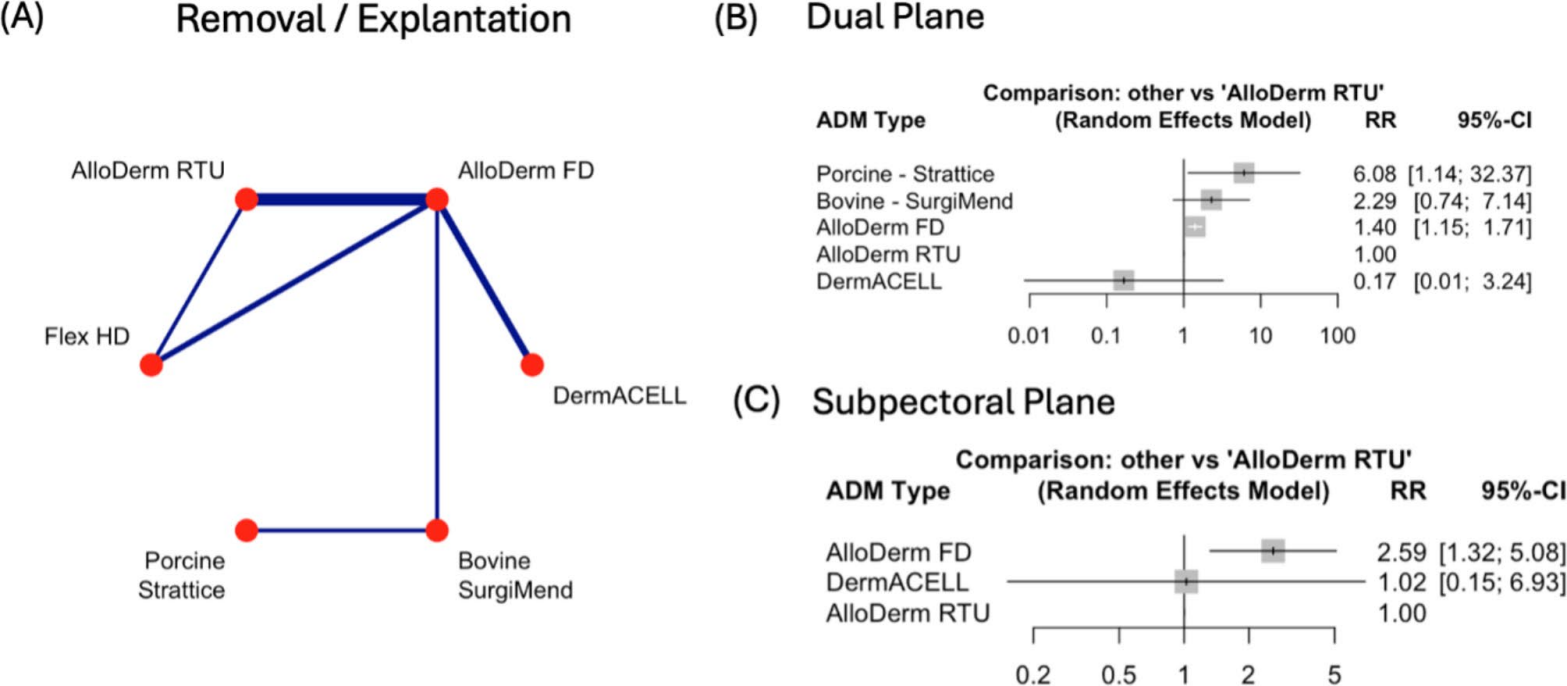
Comparison of Removal / Explantation rates across Acellular Dermal Matrices (ADMs) and surgical planes. **(A)** Network plot of pairwise comparisons between ADMs for seroma formation. Line thickness reflects the number of studies. **(B)** Forest plot of risk ratios (RR) and 95% confidence intervals (CI) comparing ADMs in the *dual plane*, with AlloDerm RTU as the reference. **(C)** Forest plot of RR and 95% CI for ADMs in the *subpectoral plane*, with AlloDerm RTU as the reference

Similarly, at the dual plane AlloDerm FD had an increased risk of 40% (RR = 1.40, 95% CI: 1.15–1.71, *p* = 0.001) and at the subpectoral plane an increase of 159% (RR = 2.59, 95% CI: 1.32–5.08, *p* = 0. 0.006). No other statistical significance was observed in the RRs when comparing the different types (Fig. 6B, C).

### Rotation

**Fig. 7:**
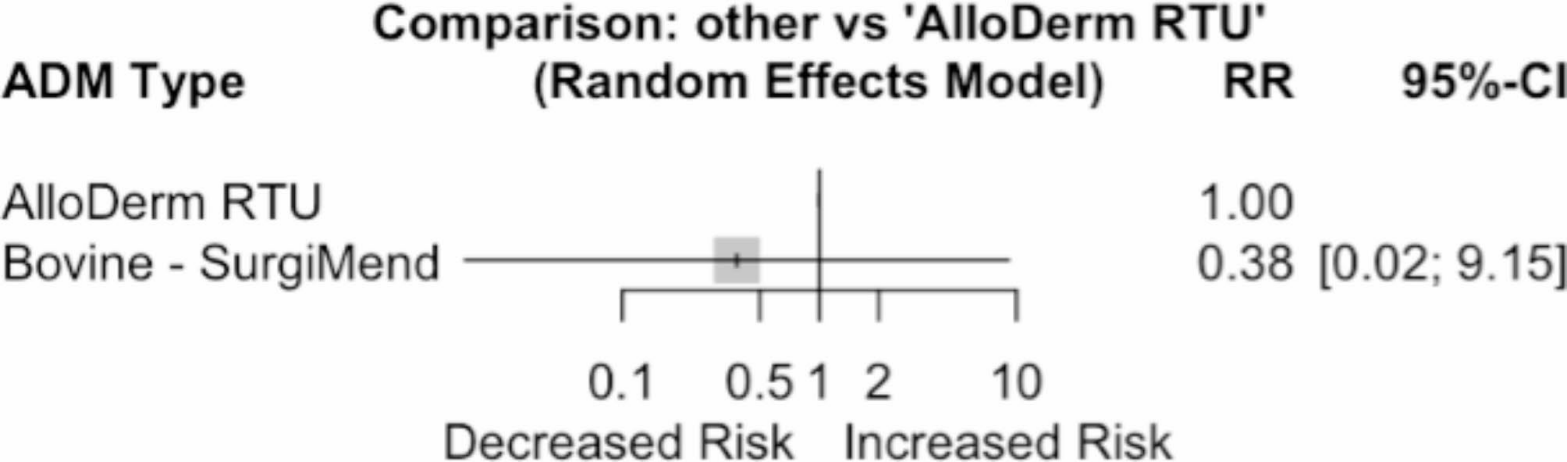
Forest plot of risk ratios (RR) and 95% confidence intervals (CI) comparing ADMs with AlloDerm RTU as the reference, for rotation rate

When comparing Bovine – SurgiMend to AlloDerm RTU, Bovine exhibited a decreased risk of rotation by 62% (RR = 0.38, 95%CI 0.02–9.15, *p* = 0.552), however, it was not statistically significant (Fig. 7). It is worth noting that while rotation rates were reported by three studies [7, 36, 37], and only Asaad et al. [36] provided rates for all arms, with the study included being of dual plane. The prevalence of rotation was 1 in 55 breasts for AlloDerm RTU and 0 in 48 breasts for Bovine treated patients.

### Infection

AlloDerm FD treated patients experienced a 40% increase in infection rates, when compared to those treated with AlloDerm RTU (RR = 1.40, 95%CIs: 1.07–1.83, *p* = 0.0148). No other statistically significant observations occurred across both plane types (Supplementary Fig. 1G).

**Fig. 8:**
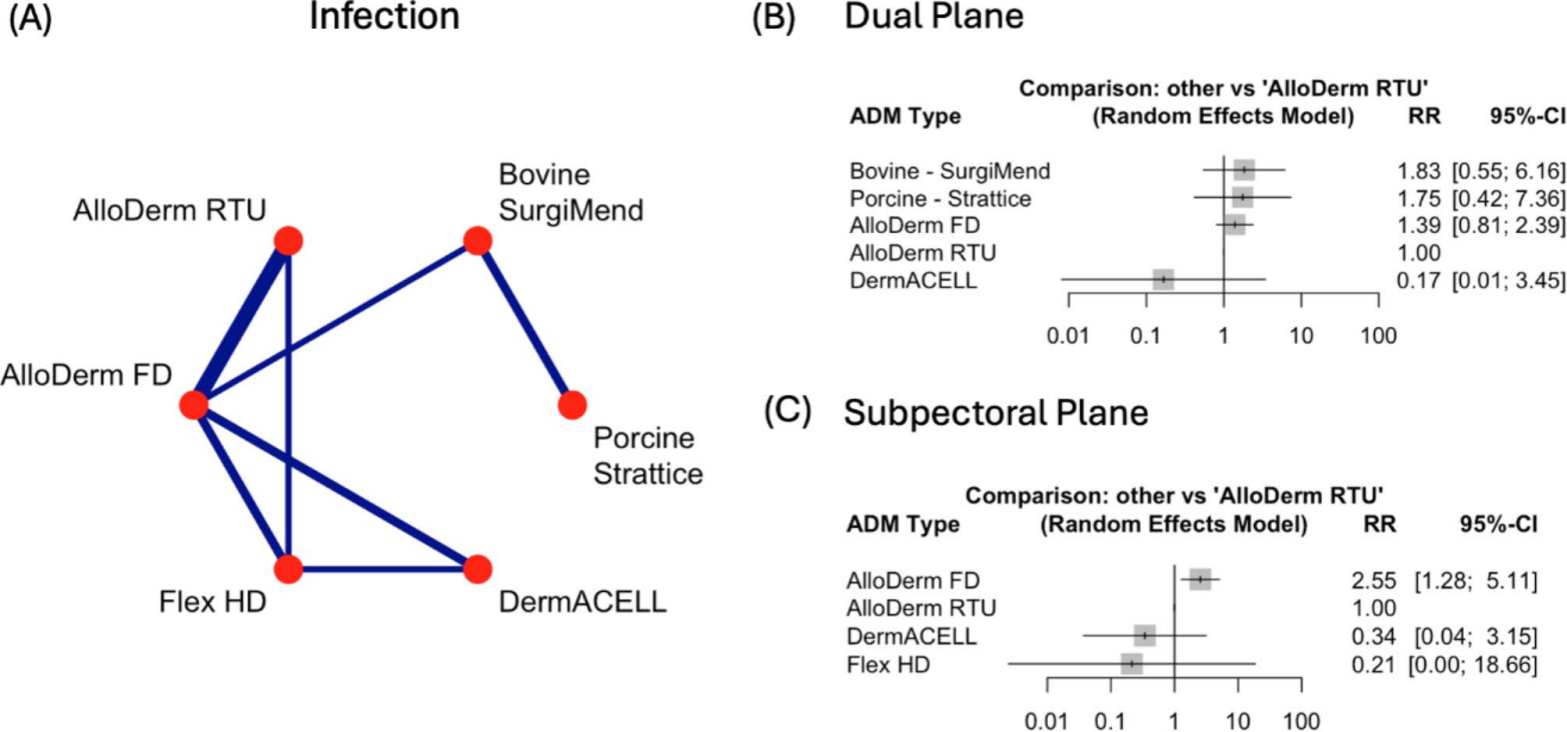
Comparison of Infection rates across Acellular Dermal Matrices (ADMs) and surgical planes. **(A)** Network plot of pairwise comparisons between ADMs for seroma formation. Line thickness reflects the number of studies. **(B)** Forest plot of risk ratios (RR) and 95% confidence intervals (CI) comparing ADMs in the *dual plane*, with AlloDerm RTU as the reference. **(C)** Forest plot of RR and 95% CI for ADMs in the *subpectoral plane*, with AlloDerm RTU as the reference

At the dual plane there were no statistically significant differences seen in the dual plane (Fig. 8B). At the subpectoral plane, when looking at AlloDerm FD versus AlloDerm RTU, an increased risk of infection was observed (Fig. 8C). More specifically, an 155% increase in risk (RR = 2.55, 95% CI: 1.28–5.11, *p* = 0.008). There were no other significant comparisons.

## Discussion

Originally developed for burn reconstruction, ADMs have seen a significant surge in popularity in the field of breast cancer reconstruction and aesthetic revision. This is due to the extensive variety of available ADM types; surgeons thus have a diverse array of options when considering ADMs in reconstruction. However, this can pose challenges in decision-making when aligning patient and procedural factors, especially in the absence of robust, comparative evidence for different types.

Prior to this review, no large-scale, comprehensive meta-analyses had compared the current evidence regarding complication and failure rates associated with all ADM types. After FDA’s increasing concerns with the high complications of ADM in immediate, two-stage subpectoral IBR, the need for more rigorous studies to establish the safety and efficacy of ADMs in breast reconstruction became evident. Our study directly addresses this by conducting a systematic review and meta-analysis of the current literature, where 91% of the included studies focused specifically on immediate IBR. This focus allows us to directly respond to the FDA’s concerns while providing a broader analysis of outcomes across different ADM types and planes of reconstruction, which has not been covered by other reviews [38]. Immediate breast reconstruction offers advantages such as larger initial volume filling of tissue expanders, reduced local tissue damage, and better support for the implant against the affected mastectomy skin [31], but it carries higher complication rates compared to delayed IBR, highlighting the need for conclusive outcome information in this area [39]. A strength of our analysis is the high quality of included studies, with most scoring 7–9 points on the Newcastle-Ottawa Scale and RCTs demonstrating robust methodologies across the board (Supplementary Tables 1, 2).

The main finding of our network meta-analysis indicates that the evaluated ADMs exhibited similar complication profiles in the context of IBR with the exception of Alloderm FD and RTU. Alloderm FD was associated with a higher risk of infection, explantation, and wound dehiscence compared to AlloDerm RTU. This finding contrasts with previous meta-analyses that reported non-significant differences or lack of superiority between these ADM types [40, 41]. It is crucial to highlight that our analysis encompassed a broader range of studies—6 to 15 studies per complication—compared to the limited 2 or 3 studies included in prior meta-analyses. Additionally, the use of a network meta-analysis enabled both direct and indirect comparisons across different ADMs, providing a more comprehensive evaluation of the relative risks associated with these products. The observed differences in complication rates between the two products can be attributed to their distinct preparation processes. AlloDerm FD is an aseptic, non-sterile freeze-dried product stored in a cryoprotective solution [42], while RTU is stored in a preservation solution (phosphate-buffered solution) and terminally sterilised by electron beam radiation [43]. These sterility differences could explain the observed differences in infection risk between the two.

Additionally, the freezing process used for the FD product leads to the formation of ice crystals, which can damage the dermal matrix, while the drying process weakens tensile strength by breaking hydrogen bonds, ultimately compromising the collagen triple-helix structure [44]. In the histological analysis by Cheon et al., higher levels of dense collagen, more red blood cells, and greater chronic inflammation were observed in the pre-hydrated ADM group. Fibrovascular ingrowth into an implanted biomaterial indicates better incorporation and suggests enhanced long-term retention without complications [45]. The enhanced angiogenesis and denser fibrocollagenous tissue observed in the biopsy results for AlloDerm RTU may contribute to better flap stability and lower risks of wound dehiscence and explantation, which align with our clinical observations [46].

Importantly, our analysis did not support the FDA’s concerns regarding higher rates of implant removal and infection for FlexHD compared to other ADMs, whether in dual-plane or subpectoral placements [11]. However, it is crucial to note that the number of studies on FlexHD was limited. Similarly, while we considered including data on AlloMax, we were unable to find any publications meeting our predefined inclusion and exclusion criteria. It is plausible that this observation may be correlated to the limited availability of research data pertaining to Allomax [47].

Lastly, in May 2023, Integra issued an immediate market recall of its bovine ADM, SurgiMend [16]; due to higher levels of endotoxins were released that exceeded the permitted levels as per the product specifications. Our study did not find any statistical differences between SurgiMend with other ADMs in any of the assessed complications.

### Seroma, hematoma

Porcine ADMs tended to show higher complication rates across multiple categories, particularly with seroma, hematoma, wound dehiscence, capsular contracture, and infection, compared to other ADM types. Among the porcine ADMs, Braxon exhibited the highest rates of seroma and hematoma and was second only to Strattice in wound dehiscence. Given that Braxon is typically used in pre-pectoral reconstructions [48], and systematic reviews indicate no significant difference in complication rates between pre-pectoral and subpectoral [49] or dual-plane reconstructions [50], such complications might be inherent to the Braxon ADM utilised. Upon direct comparison of Porcine ADMs with all other ADMs, no significant differences were observed. However, the inclusion of only two studies in the analysis limits the ability to detect true differences between ADMs, suggesting that the lack of significant findings may be due to insufficient data rather than the absence of actual differences. Further comparative studies are needed to obtain more conclusive results.

Several studies have indicated that seroma and hematoma in IBR are non-significant when ADM is used or not [51–53]. Our current results feed into this, showing that the risk of seroma and hematoma formation is not significantly influenced by the type of ADM used or whether ADM is employed at all in comparison to submuscular approaches. For such surgeries, emphasis in reducing complications should perhaps shift more towards surgical technique, patient selection, and pre/post-operative care, rather than solely focusing on the type of ADM used.

When it came to hematoma, although none of the comparisons reached statistical significance, Bovine (SurgiMend) demonstrated a trend towards a lower risk compared to Porcine (Strattice), with the upper bound of the CI approaching 1.0 (RR = 0.21, 95% CI: 0.04 to 1.02). Given the low number of breasts in this subtype (*n* = 100), a larger sample size could potentially reveal a difference that is clinically or statistically relevant.

### Wound dehiscence and capsular contracture

Porcine ADMs exhibited the highest rates of wound dehiscence and capsular contracture, while bovine ADMs showed the lowest wound dehiscence and zero capsular contracture. This difference can be attributed to the superior mechanical properties of bovine ADMs. The study by Adelman et al. reported that bovine ADM had nearly double the ultimate tensile strength, suture retention strength, and tear resistance compared to porcine ADMs [54]. These qualities make bovine more suitable load-bearing applications, potentially reducing complications like dehiscence and capsular contracture. However, it is important to note that the study’s author was affiliated with TEI Biosciences.

Additionally, the average follow-up of the studies was highly heterogeneous with most ranging from 12 to 24 months, which might have not been sufficient to fully capture the development of capsular contracture, which typically requires longer observation periods [55]. Despite observed trends, the absence of statistical significance in the NMA upon Porcine and Bovine, suggests that the differences are not currently robust enough to guide clinical decision-making.

### Limitations

Limitations of existing literature include small sample size, potential biases such as single-surgeon variation and industry affiliations. Furthermore, the reporting of outcomes and complications lacks uniformity and precise definitions, resulting in significant discrepancies among research papers. For example, many studies do not distinguish between minor and major infections, making it difficult to assess the true severity and clinical relevance of reported rate [16]. Additionally, several studies did not explicitly indicate the type of Alloderm ADM used, resulting in its classification as “Unspecified,” with the possibility of it belonging to either the FD or RTU category. To avoid skewing of results, the meta- analysis only included data from studies where AlloDerm FD or RTU was specifically reported to ensure results can be clinically translated.

Furthermore, it is important to note that the observed differences may be influenced by several confounding variables not accounted for in this meta-analysis. Patient characteristics such as high BMI, smoking, preoperative radiotherapy, advanced cancer staging, and large breast cup size (over D) are all known to increase the risk of complications, suggesting that differences observed might be inherent to the patient cohort rather than the ADMs themselves [25, 33, 56, 57].

Similarly, practice variability, including surgeon experience, incision technique (skin sparring versus nipple sparring) [58–60], and whether axillary node dissection was performed [61, 62] were not controlled for in this analysis. Additionally, the preparation method of ADMs is another important factor. AlloDerm FD requires a rehydration step that can take up to 40 min depending on the matrix thickness, while AlloDerm RTU is ready-to-use thus no need for rehydration. This variability in rehydration technique could impact clinical outcomes [63]. Lastly, Finkelstein et al. showed that ADM thickness can directly affect reconstructive outcomes and complications, with medium-thickness ADMs having fewer overall incidents of wound dehiscence compared to patients with thicker ADMs [64]. Finkelstein et al. highlighted that ADM thickness can directly influence reconstructive outcomes and complication rates, with medium-thickness ADMs showing fewer instances of wound dehiscence compared to thicker ADMs [64].

Several studies lacked comprehensive reporting of patient demographic data, including BMI and smoking status, as well as details regarding therapeutic interventions such as chemotherapy and radiotherapy, along with their timing in relation to surgical procedures. These variables are well-established predictors of operative outcomes, highlighting the importance of complete data reporting to enable robust and informed decision-making. Future studies should conduct a meta-regression analysis to detect the influence of these factors, as well as inclusion of cancer as an outcome.

Multiple studies included in this meta-analysis reported conflicts of interest, with financial ties to companies involved in the manufacturing or selling of ADMs (Supplementary Table 3). These conflicts primarily involved consultancy roles, research funding, and equity stakes, which could introduce potential bias in the outcomes or interpretations presented in those studies.

## Conclusion

This systematic review and network meta-analysis revealed no significant differences in complication rates across ADM types used in IBR, apart from increased risk of infection, explantation, and wound dehiscence for AlloDerm FD over AlloDerm RTU. Absence of statistically significant differences between other ADM subtypes, with the exception of Alloderm FD, indicates that the choice of ADM may not significantly impact overall complication rates in most clinical scenarios. Nevertheless, heterogeneity in definitions reported outcomes as well as lack of adjustment for patient demographics, surgical techniques, and surgeon experience, may have contributed to the observed results. Further high-quality, long-term, double-arm studies are warranted to provide more definitive evidence regarding the comparative complication profile of specific ADMs.

## Electronic supplementary material

Below is the link to the electronic supplementary material.

Supplementary Material 1: Table 1. Quality appraisal of studies the Newcastle-Ottawa Scale. Table 2. Quality appraisal of RCTs using the CONSORT 2010 checklist. Table 3. Conflict of Interest reported under each study. Fig. 1. Forest Plots representing the risk ratio (RR) and confidence intervals (CIs) for (A) Seroma, (B) Haematoma, (C) Wound Dehiscence, (D) Capsular Contracture, (E) Explantation / Removal, (F) Rotation, (G) Infection. Alloderm* represents Alloderm Unspecified, which is undefined.

## Data Availability

No datasets were generated or analysed during the current study.
