# Supplementary figures and images for: A comparison of acellular dermal matrices (ADM) efficacy and complication profile in women undergoing implant-based breast reconstruction: a systematic review and network meta-analysis
Source: BMC Cancer. 2024 Dec 31;24:1598. doi: 10.1186/s12885-024-13359-3 (PMC11686910; doi:10.1186/s12885-024-13359-3)

(A) Seroma

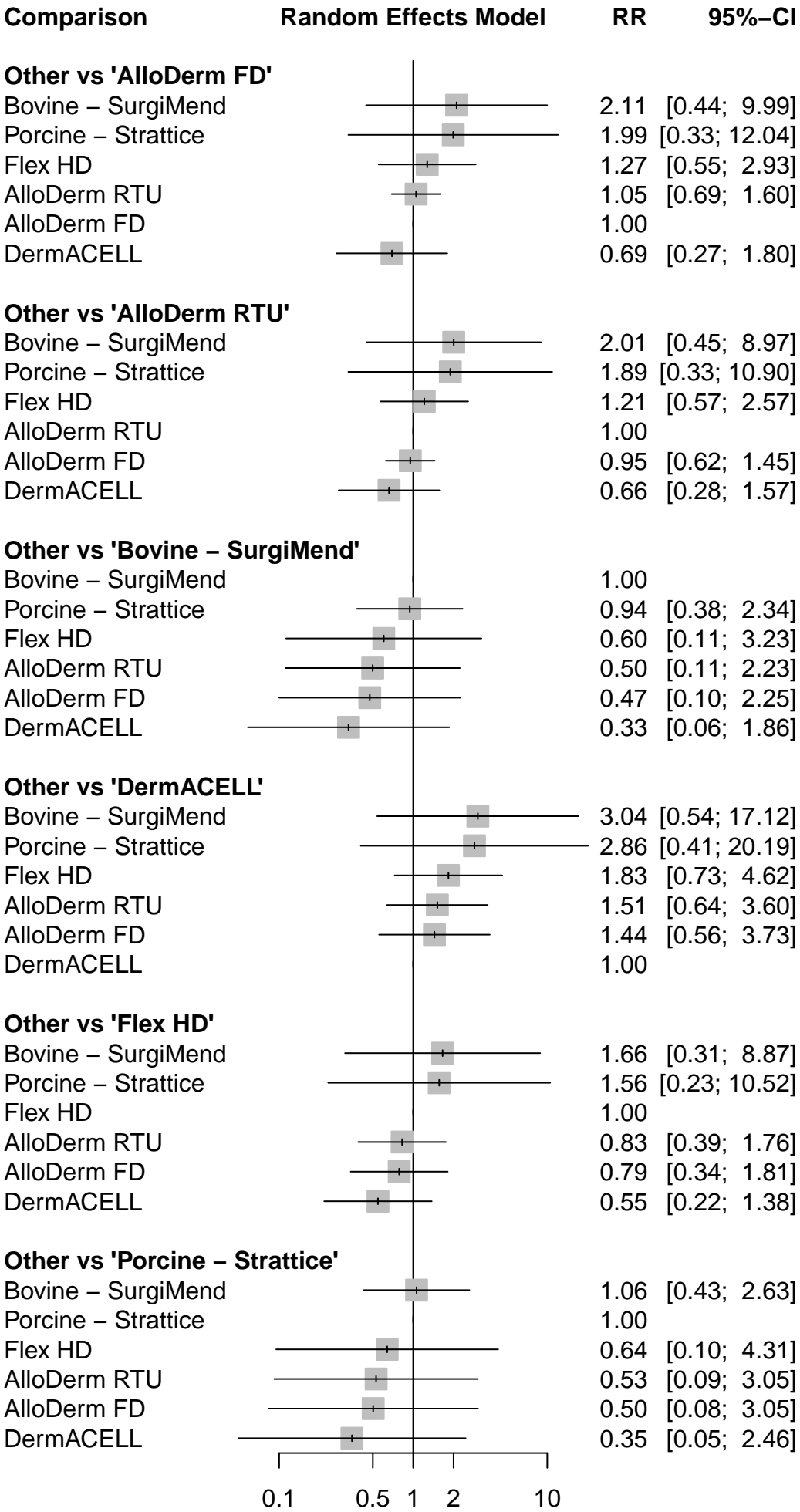

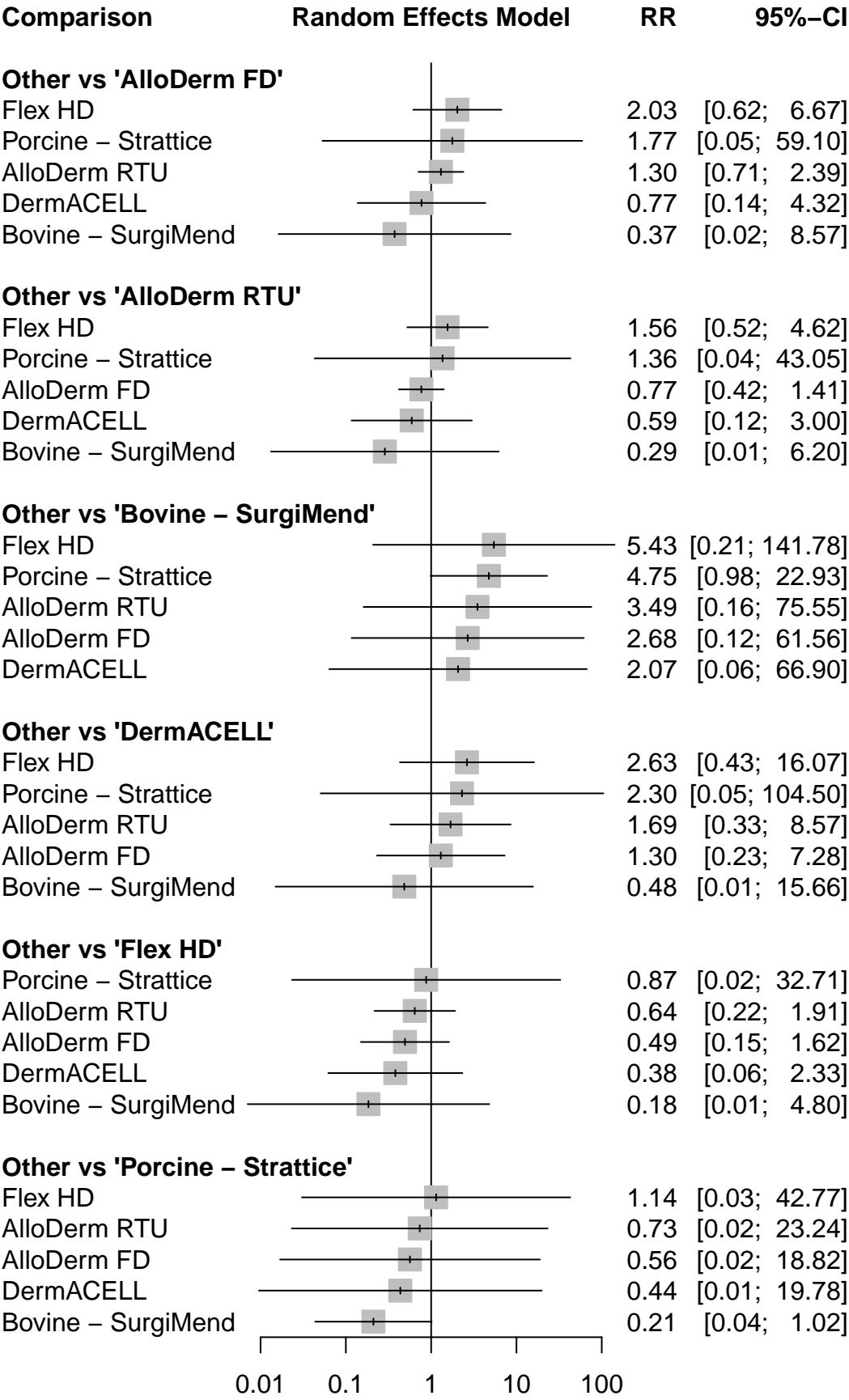

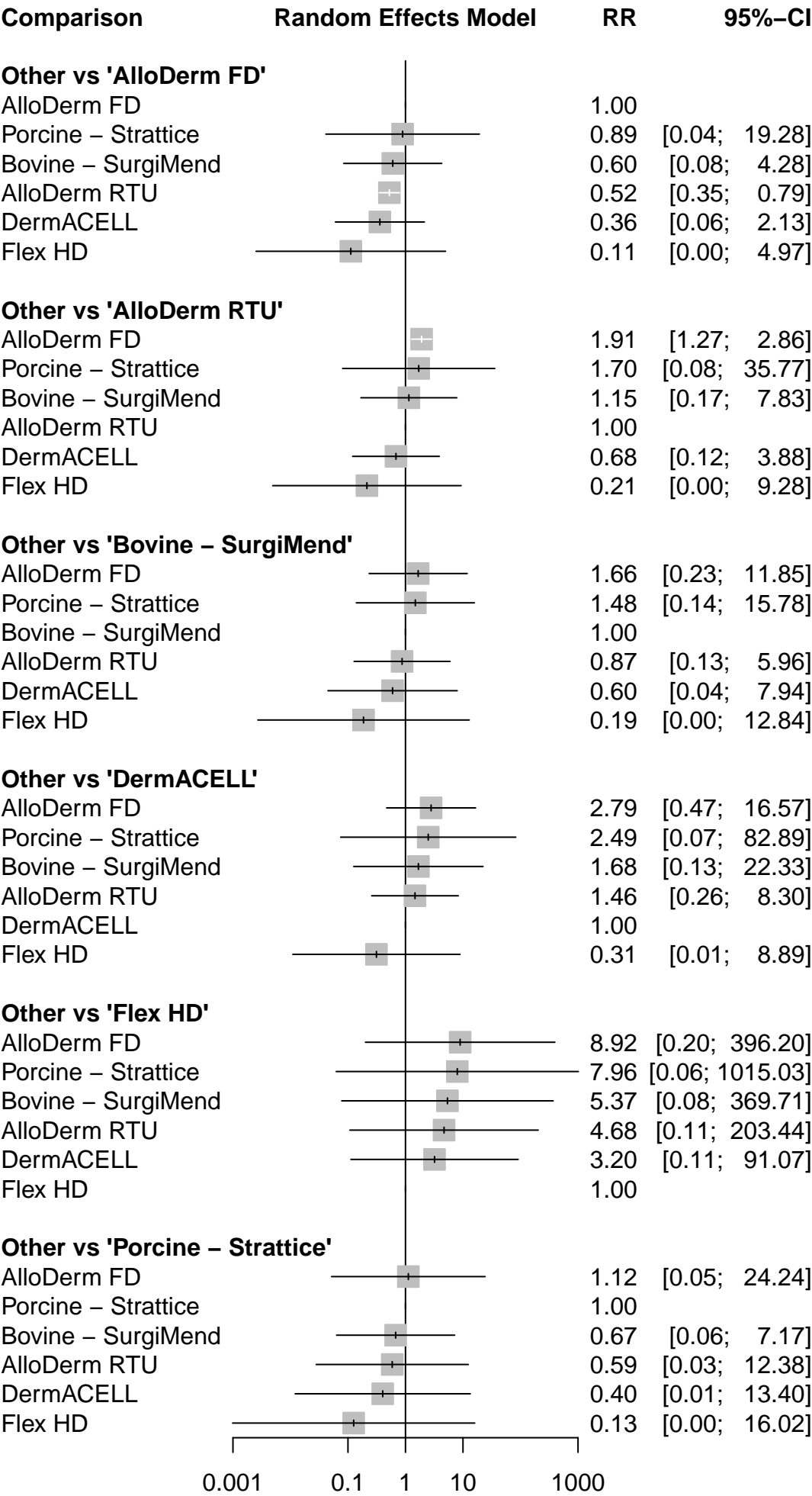

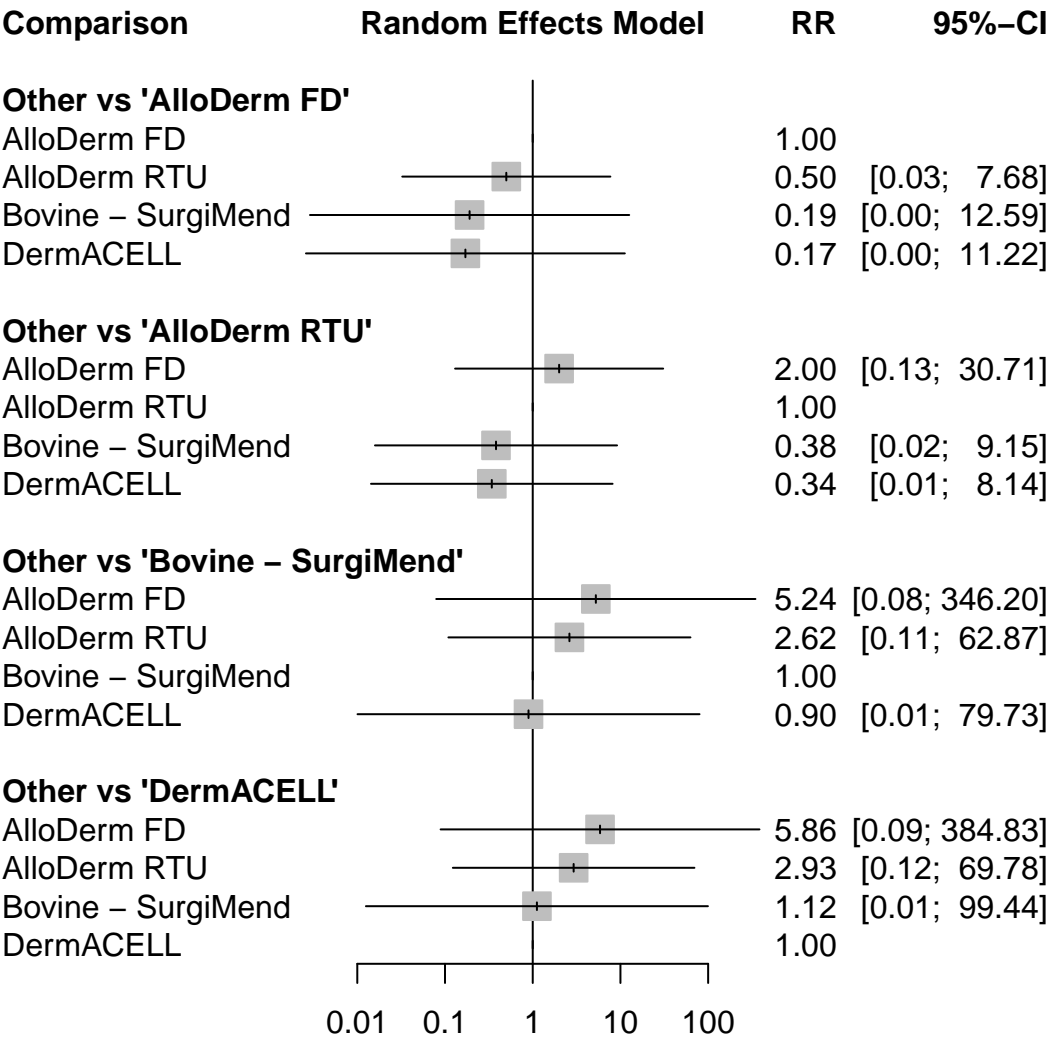

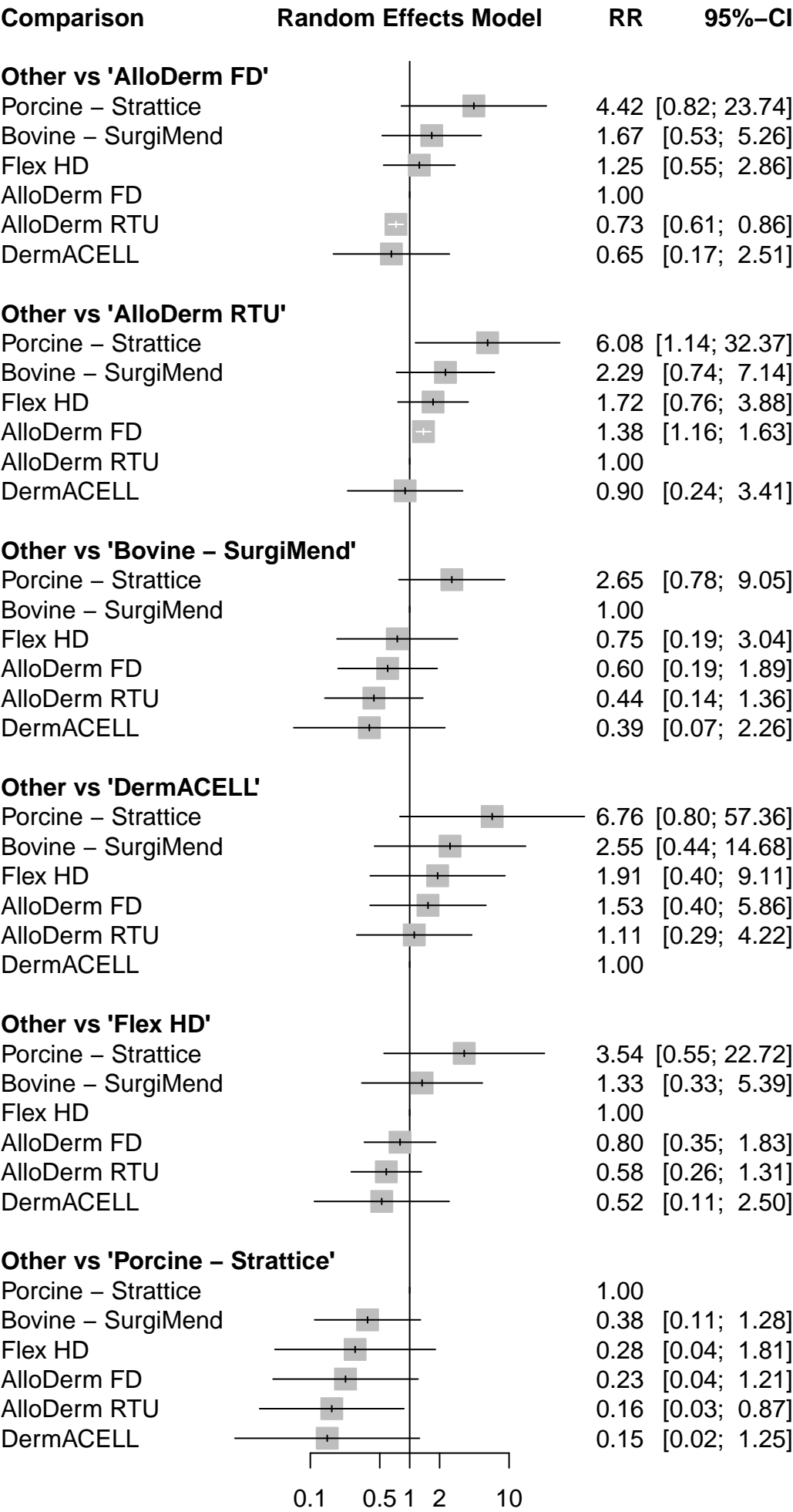

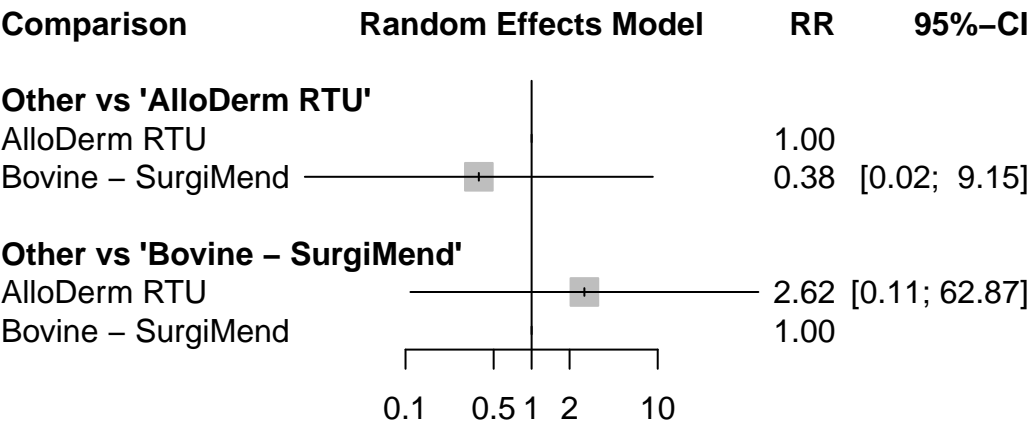

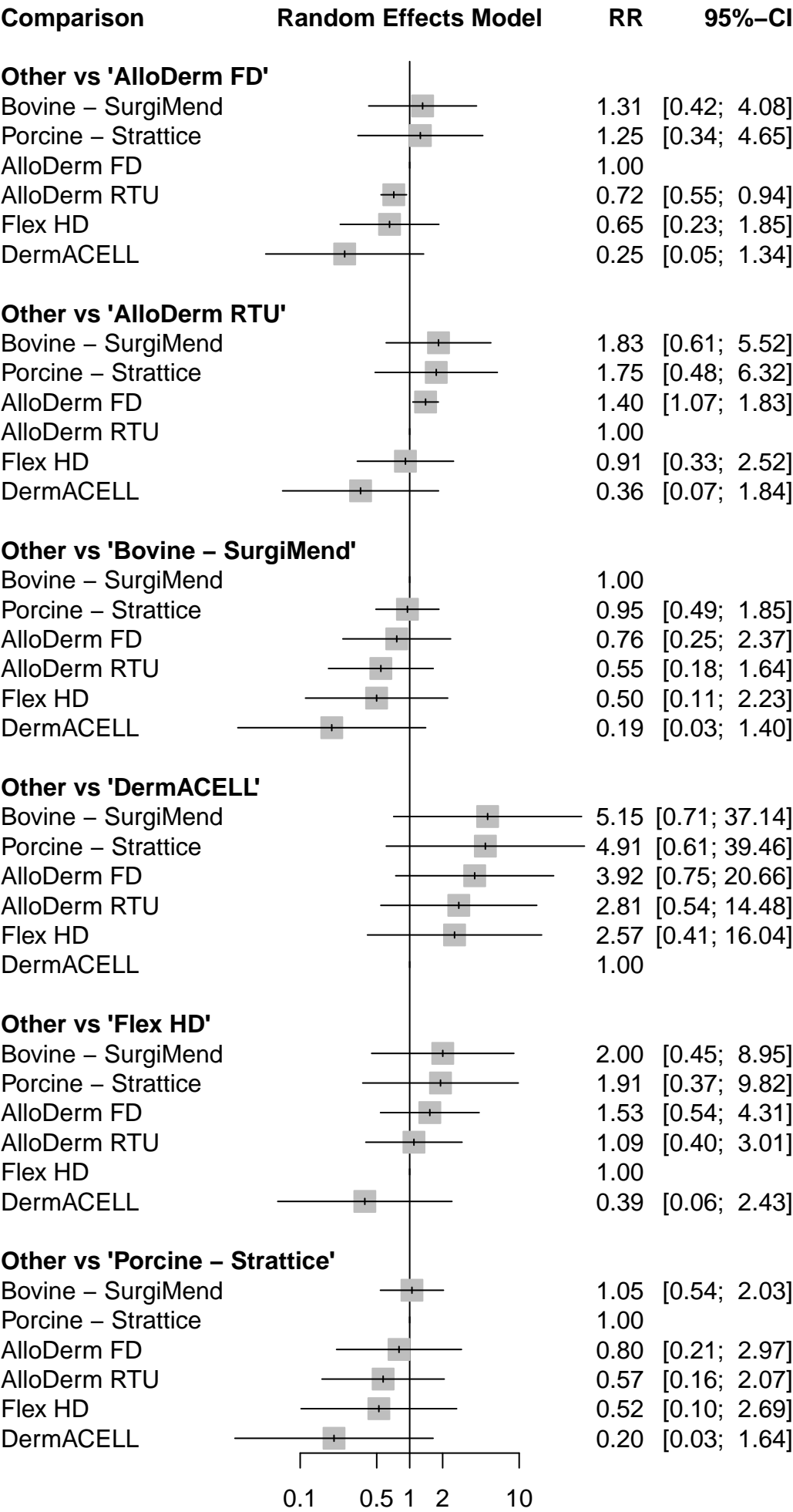

Supplement: Supplementary file 1 — Supplementary Material 1: Table 1. Quality appraisal of studies the Newcastle-Ottawa Scale. Table 2. Quality appraisal of RCTs using the CONSORT 2010 checklist. Table 3. Conflict of Interest reported under each study. Fig. 1. Forest Plots representing the risk ratio (RR) and confidence intervals (CIs) for (A) Seroma, (B) Haematoma, (C) Wound Dehiscence, (D) Capsular Contracture, (E) Explantation / Removal, (F) Rotation, (G) Infection. Alloderm* represents Alloderm Unspecified, which is undefined. [file 12885_2024_13359_MOESM1_ESM.pdf]
